# Space Physiology and Technology: Adaptations, Countermeasures, and Opportunities for Wearable Systems

**DOI:** 10.34133/cbsystems.0477

**Published:** 2026-04-17

**Authors:** Shamas U.E. Khan, Rejin J. Varghese, Panagiotis Kassanos, Dario Farina, Etienne Burdet

**Affiliations:** Department of Bioengineering, Imperial College London, London W12 0BZ, UK.

## Abstract

Space poses substantial challenges for humans, leading to physiological adaptations in response to an environment vastly different from Earth. A comprehensive understanding of these physiological adaptations is necessary to develop effective countermeasures that support human life in space. This narrative review first focuses on the impact of the space environment on the musculoskeletal system. It highlights the complex interplay between bone and muscle adaptations and their implications on astronaut health. Despite advances in current countermeasures, such as resistive exercise and pharmacological interventions, they remain partially effective, bulky, and resource-intensive, posing challenges for future missions aboard compact spacecraft. This review proposes wearable sensing and robotic technologies as promising alternatives to overcome these limitations. Wearable systems, such as sensor-integrated suits and (soft) exoskeletons, can provide real-time monitoring, dynamic loading, and exercise protocols tailored to individual needs. These systems are lightweight, modular, and capable of operating in confined environments, making them ideal for long-duration missions. In addition to space applications, wearable technologies hold considerable promise for terrestrial uses. They could support rehabilitation and assistance for the aging population and individuals with musculoskeletal disorders, and enhance physical performance in healthy users. By integrating advanced materials, sensors, actuators, and intelligent, energy-efficient control, these technologies can bridge gaps in current countermeasures while enabling broader applications on Earth.

## Introduction

**Fig. 1.**
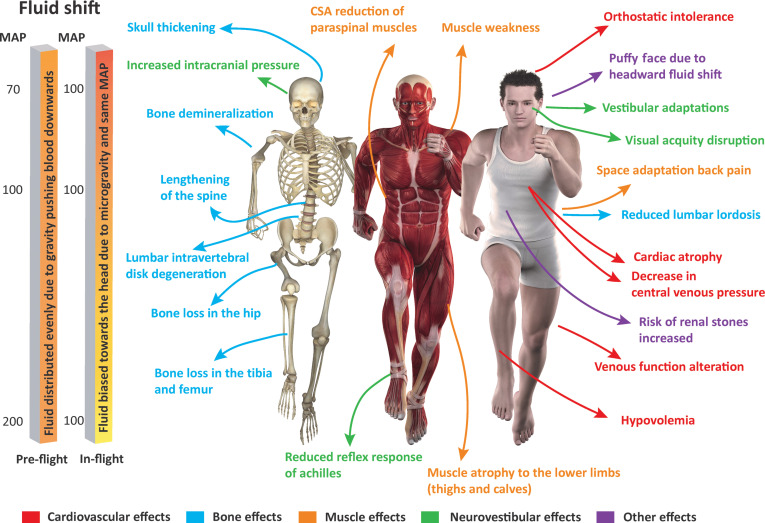
Physiological adaptations due to microgravity in outer space. Parts of the figure have been modified from Ref. [305] and Shutterstock.com (license: 182406275).

Human physiology continually adapts to Earth’s gravitational field from the earliest stages of embryonic development. As a result, gravitational unloading in outer space causes adverse effects that challenge human space exploration and habitation [1]. Frequent space missions and experiments at the International Space Station (ISS) (orbiting 300 to 435 km above Earth) have provided extensive data on the physiological challenges faced by astronauts [2] (Fig. 1). As astronauts ascend from Earth, a headward bodily fluid shift occurs [3], causing changes in body fluid distribution and electrolyte homeostasis. Muscle atrophy and bone resorption are notable concerns during prolonged space missions. Early space missions lasting just a few days resulted in muscle atrophy of up to 16% despite countermeasures [4]. Bone resorption occurred at a rate of 1% to 2% per month, and although researchers partially mitigated the effects with countermeasures, the deconditioning was not entirely opposed.

The human musculoskeletal system enables locomotion, postural control, and the performance of activities of daily living (ADL). In space missions, adverse effects on this system can impact task performance, increase long-term risks, and necessitate intensive post-flight rehabilitation [4]. Astronauts may often travel to space for several months, and the adaptations observed in human physiology post-flight resemble those of aging on Earth [5]. With more extended space missions (and prolonged exposure to radiation and microgravity), the risk becomes more substantial and could even prove fatal if not mitigated. It is uncertain how long life can be sustained in microgravity, but effective countermeasures to space-related challenges are crucial for safe, extended space travel.

Through this review, we discuss the challenges of the outer space environment and the musculoskeletal health complications derived from prolonged exposure. The currently employed solutions to address these complications are discussed, along with their limitations and potential solutions. The paper is organized as follows:

• The “Outer Space Stressors” section discusses various aspects of outer space that act as stressors impacting the musculoskeletal system.
• The “Musculoskeletal Adaptations and Injuries in Space” section describes the physiological adaptations and musculoskeletal changes that occur due to these stressors during long-duration space missions.
• The “Current Countermeasures” section reviews currently deployed, research-based countermeasures adopted by various space agencies and laboratories, along with their limitations.
• The “The Opportunities and Challenges for Wearable Technologies as Countermeasures” section highlights the opportunity for wearable robotic and sensing technologies as a potential alternative to current exercise-based countermeasures, along with recent examples and developments in the literature.
• The “Discussion and Perspectives for Future Research and Development” section concludes with key takeaways, a critical analysis of the limitations of current studies, and recommendations for future research and innovation.

## Outer Space Stressors

Outer space represents a challenging environment with unique stressors. The most prominent stressors are prolonged microgravity exposure, radiation exposure, and psychosocial stressors, which have profound adverse effects on the human body and performance [6] (Fig. 1). As space missions are planned to stretch onto Mars and beyond, understanding these stressors becomes increasingly essential, as astronauts will be longer in space. This understanding can guide the development of novel countermeasures to mitigate physiological deconditioning, improve astronaut performance, and mission success.

### Weightlessness/microgravity

Weightlessness and microgravity are used interchangeably. However, microgravity refers to environments where gravitational forces are present but remarkably reduced, and weightlessness refers to the sensation or condition of not feeling any weight. In orbit, the gravitational force continues to act on an object, such as a satellite or the ISS. However, as it travels at very high speeds, the forward force balances the gravitational pull, allowing the object to maintain a relatively constant height in its curved orbit. This results in continuous freefall, causing the inhabitants to experience weightlessness [7,8].

On Earth, our bodies have adapted to 1G gravity, which ensures the uniform distribution of bodily fluids crucial for homeostasis and mean arterial pressure regulation [3]. Microgravity, however, induces a cephalic fluid shift, removing hydrostatic pressure from tissues, muscles, and bones, leading to deconditioning (as graphically illustrated in Fig. 1). The musculoskeletal system is among the most affected systems by microgravity. Anti-gravity muscles, such as the soleus, gastrocnemius, quadriceps femoris, spinal postural muscles, and leg extensors, are vital for posture, balance, and movement on Earth. Microgravity mechanically unloads these muscles, inhibiting mechanotransduction signaling for protein synthesis [9]. Consequently, muscle atrophy, particularly in anti-gravity muscles, and bone resorption occur by promoting osteoclastic activity and disrupting calcium homeostasis. Upon returning to Earth, astronauts must undergo intensive rehabilitation to re-adapt to Earth’s environment and counter bone resorption and muscle atrophy experienced during spaceflight.

### Radiation

**Fig. 2.**
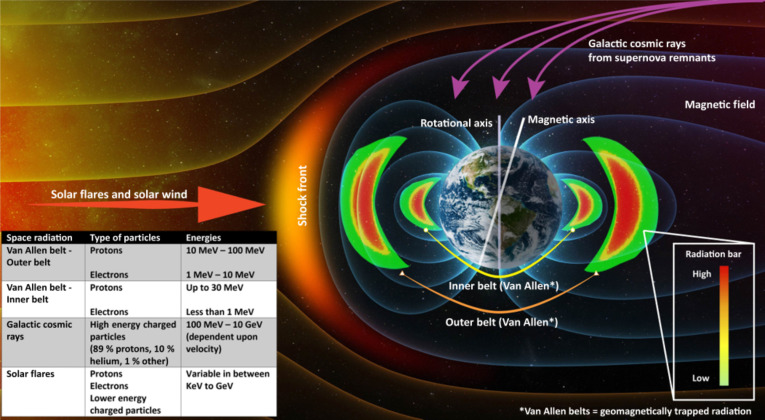
Space radiation with their respective energies. Parts of the figure have been modified from Shutterstock.com (license: 121554235).

Ionizing space radiation, including x-rays, gamma rays, and other high-frequency waves, presents substantial health risks to humans during space travel, requiring cautious planning and safety countermeasures, especially for interplanetary or moon expeditions [10–12]. Earth’s magnetic field partially protects the ISS [13], but it is still susceptible to geomagnetically trapped radiation, galactic cosmic rays (GCRs), and solar flares (Fig. 2). Ionizing radiation, whether acute or chronic, can cause short- and long-term adverse effects on the human body. Its ability to penetrate the biological tissues can damage DNA, increase free radicals in the body, and increase oxidative stress, consequently heightening the risk of conditions like cancer or central nervous system (CNS) disruptions [14]. Animal studies show increased cancellous bone loss and higher osteoclasts (bone-resorbing cells) [15,16].

The average annual radiation exposure to humans in the United States is approximately 6.2 mSv. During a 6-month ISS mission, crew members experience exposure to radiation levels between 50 and 100 mSv, with higher levels during extravehicular activities (EVAs) [17]. It is predicted that a mission to Mars may result in over 1,000 times the annual exposure on Earth. More than 1 Sv of ionizing radiation can cause acute symptoms and potentially have fatal consequences [15].

Besides its biological implications, ionizing radiation in space can also affect spacecraft equipment and instrumentation. Radiation can induce single-upset events, material degradation, or latch-ups, thereby affecting system reliability. Manifestations depend on several factors, such as mission parameters, shielding, individual sensitivity, and absorbed dose [18,19]. Therefore, management and countermeasures are crucial for maintaining physiological and system functioning and integrity during deeper and longer space missions.

### Psychosocial stressors

Astronauts face psychosocial stressors such as isolation, confinement, disrupted communication, and interpersonal conflicts that substantially impact their mental health and overall well-being. The psychological stress can affect cognition, attention, and mindfulness during prolonged space missions [6,20,21]. Individual responses to stress vary, making cognitive appraisal a determinant of psychological stress and its consequences [22]. Psychosocial and physical stressors are linked, with each potentially worsening the other in a dynamic, reciprocal relationship, hence resulting in musculoskeletal, cardiovascular, and neurological adverse manifestations. An increased state of psychosocial stress triggers a neuroendocrine response that dysregulates the hypothalamic–pituitary axis (HPA), resulting in increased body cortisol and catecholamines, suppressing osteoblastic activity and, subsequently, increasing bone resorption and muscle protein degradation [23–26]. Therefore, addressing these stressors is imperative for sustaining psychological resilience throughout extended space missions.

### Effects of outer space stressors on the musculoskeletal system

While outer space stressors induce adverse physiological effects across several body systems, this review focuses on the adverse impact on the musculoskeletal system. This is justified as the immediate decline in this system can critically impair astronaut mobility, performance, and post-mission recoverability. Table 1 provides an overview of outer space stressors and their effects on the musculoskeletal system. This comparison aids in developing countermeasures to mitigate and prevent such effects from outer space stressors.

**Table 1.** Effects of spaceflight stressors on the musculoskeletal system

Stressor	Mechanism of impact	Effects on the musculoskeletal system
**Microgravity**	- Mechanical unloading reduces gravitational load on bones and muscles [9] - Alters neuromuscular coordination and proprioception [45] - Disruption of the calcium homeostasis [9]	- Atrophy of antigravity muscles (e.g., soleus and gastrocnemius) - Decreased muscle fiber CSA - BMD loss, especially in weight-bearing regions (e.g., spine and pelvis)
**Radiation**	- Oxidative stress via reactive oxygen species and free radicals [14] - Causes DNA damage and cellular senescence [14] - Causes chronic inflammation and hinders stem cell function [14]	- Muscle degradation and bone resorption through increased protein breakdown and impaired repair mechanisms - Bone loss due to decreased osteoblast activity and increased osteoclast activity
**Psychosocial**	- Prolonged psychological stress elevates cortisol by disrupting HPA axis and other catabolic hormones [23–26] - Affects sleep, recovery, and neuroendocrine balance [23–26] - Altered motivation for physical activity [20]	- Muscle catabolism due to elevated cortisol levels - Reduced motor control and coordination - Disuse atrophy from decreased physical engagement or motivation - Poor musculoskeletal adaptation

## Musculoskeletal Adaptations and Injuries in Space

This section examines the effects of the various stressors discussed in the previous section on the musculoskeletal system, focusing on physiological adaptations and associated injuries.

### Human locomotion and movement

**Fig. 3.**
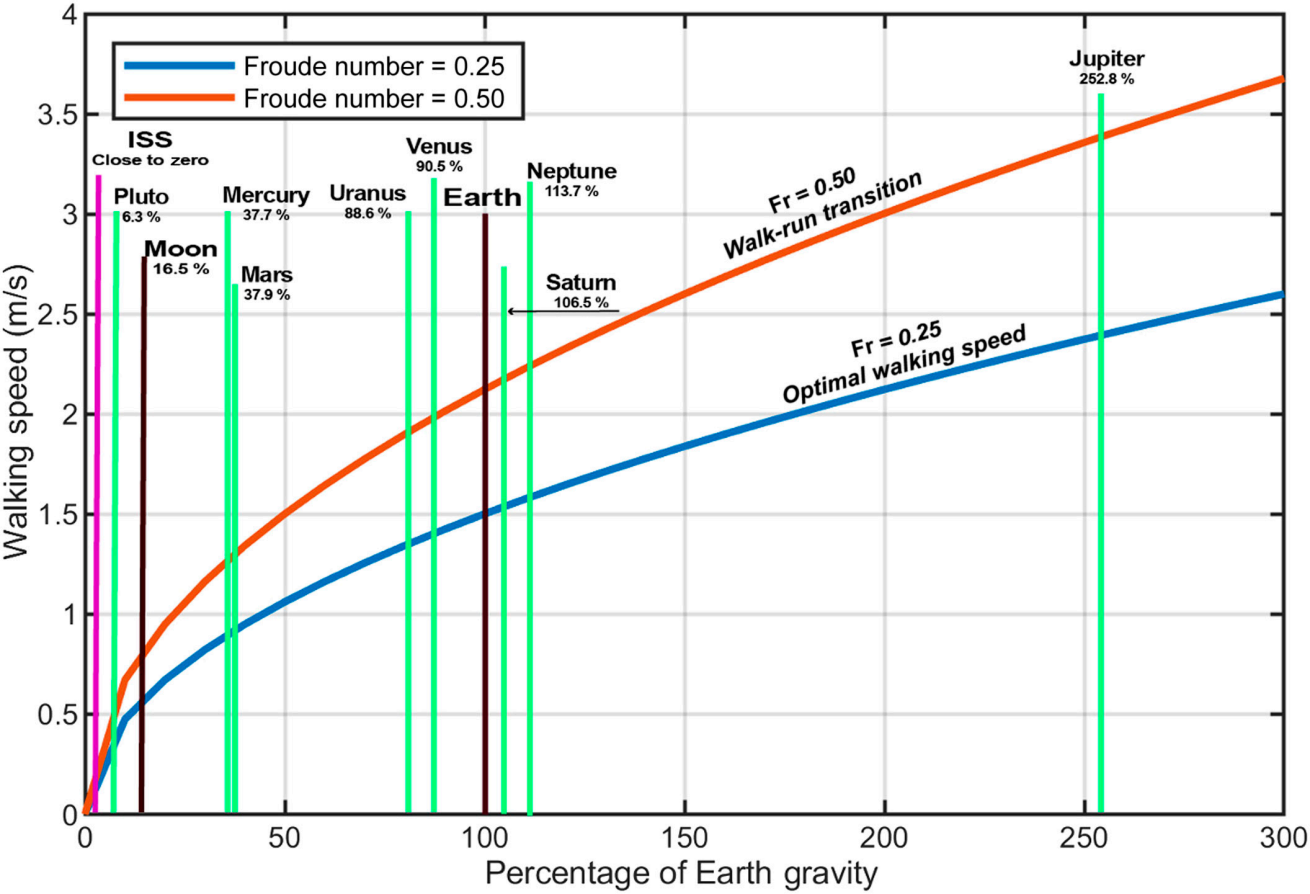
Simulated different optimal walking speeds as a function of gravity. The figure was generated from data in Ref. [29].

Human locomotion on Earth has evolved and adapted in response to Earth’s gravity [27]. In space, the reduced ground reaction forces (GRFs) result in decreased muscle force, affecting normal human locomotion [28]. Stride length and walking speed are influenced by gravity. A Froude number (which is the ratio of inertial to gravitational forces or kinetic to potential energy = Fr *= v*^2^/*gL*, where *v* is velocity, *g* is the gravitational acceleration, and *L* is leg length) of 0.25 and 0.50 corresponds to optimal walking speed and walk-to-run transition on Earth, respectively [29–34]. In reduced gravity, the optimal walking speed and walk-to-run transition decrease (Fig. 3) [29]. Humans in lower gravity (e.g., on Mars) may prefer running at lower velocities. On the moon, astronauts tend to hop rather than walk. This strategy minimizes metabolic costs and increases efficiency and stability [34–37].

GRFs, influenced by gravitational acceleration, are lower in partial gravity or microgravity. Schaffner et al. [38] and Genc et al. [39] reported that GRF decreases under reduced load and increases at higher speeds, but was significantly lower than on Earth (46% and 25%, respectively). A reduction in bone mineral density (BMD) may also have contributed to a decrease in GRF. Exercise devices and protocols on the ISS are designed to prevent BMD loss, but they usually do not induce forces comparable to those on Earth [40,41]. Current countermeasures on board in space systems have not yet provided regular forces equivalent to Earth’s 1G (= 9.8 m/s^2^) gravitational pull [42,43].

A different aspect of locomotion, stability, depends on head and gaze coordination with sensory and motor information from the CNS [44]. In microgravity, the vestibular–ocular system unloading affects balance and gaze control, leading to disorientation [45,46]. Post-spaceflight, astronauts face an increased risk of tipping [47,48]. Disturbances in gaze control, locomotion, posture, muscle atrophy, and bone resorption can result in long-term health implications or injuries [49]. Locomotion and movement during EVAs or intravehicular activities (IVAs) have resulted in musculoskeletal injuries and traumas to extremities, back, and neck [50]. Further research is needed to investigate how additional spaceflight stressors, such as psychosocial factors and radiation, impact human locomotion and movement. While the mechanical and physiological aspects of locomotor adaptation in microgravity have received notable study, the effects of these stressors are still unclear. This gap underscores the need for a more comprehensive approach to developing countermeasures that account for the interplay among environmental, psychological, and biological factors.

### Spine and back

On average, in microgravity, the human spine extends by 4 to 7 cm [4]. Spinal lengthening is believed to induce tension in the dorsal nerve roots of the lumbar spine [51], which can lead to back pain, commonly referred to as space adaptation back pain (SABP). Wing et al. [52] reported that 14 of 19 astronauts experienced moderate lower back pain accompanied by a 2.1-cm increase in spine height within the first 3 days of spaceflight. These findings were confirmed by a head-down tilt bed rest (HDBR) experiment, a ground-based model that mimics Earth’s microgravity conditions [53]. SABP impairs astronauts’ mood, concentration, and performance. Adopting the knee-to-chest position, or “fetal tuck”, has been found to alleviate back pain [54–57]. Back pain relief is also observed following in-space exercises and analgesic medications [58]. Astronauts also have a 4 times higher risk of herniated nucleus pulposus compared to non-astronauts (data from 983 healthy non-astronauts and 321 astronauts after spaceflight, acquired from the Longitudinal Study of Astronaut Health Database [59]).

Magnetic resonance imaging and ultrasound-based studies (performed pre-flight, immediately, and 30 days post-flight) reported reduced spinal muscle cross-section area (CSA), decreased lumbar lordosis, reduced bone mass, muscle weakness and para-spinal muscle reduction/atrophy, increased spine stiffness, widespread spinal microfractures, and inter-vertebral disc (IVD) degeneration [54–68]. Astronauts in studies [66–68] underwent countermeasures that may have influenced the differences observed between the lumbar and cervical regions; however, muscle atrophy was also noted in the lumbar IVD.

### Muscle and bone atrophy

**Fig. 4.**
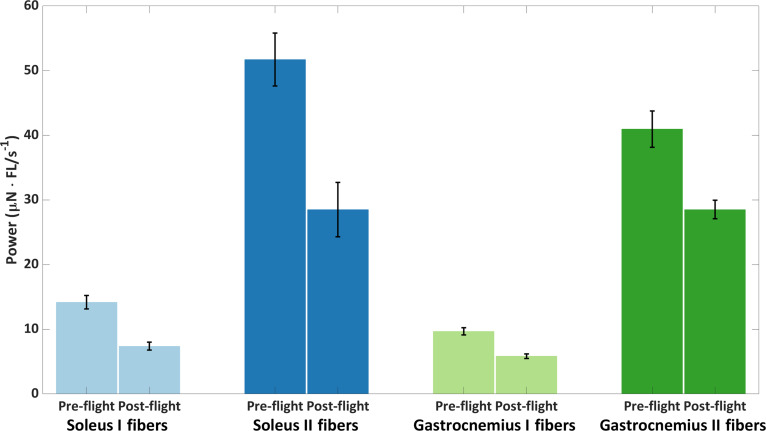
Muscle atrophy by fiber type of soleus and gastrocnemius measured at maximal contraction force in pre-flight and post-flight of 10 astronauts (data from Ref. [80]).

In 1962, Mohler [69] first raised concerns about muscle atrophy in astronauts. NASA’s Skylab experiments in 1975 documented muscle atrophy in crew members and explored potential countermeasures to mitigate it [70,71]. Biostereometric measurements conducted on the lower limb (gastrocnemius and soleus) and upper limb (biceps brachii and brachioradialis) muscles revealed nonsignificant muscle loss in the arms but significant muscle loss in the trunk and lower limbs [70,72–74]. Calves experienced more atrophy than thighs [75–77], with a reduced reflex response in the Achilles tendon reported during the Skylab 3 and 4 missions [78]. The soleus muscle was most affected by atrophy, with muscle loss observed even in short space missions [75,76]. Ground models that mimic microgravity in space, such as HDBR, limb immobilization, or water immersion, also reported muscle atrophy and bone resorption at lower rates than in space [79]. Muscle atrophy in the soleus and gastrocnemius fiber types is illustrated in Fig. 4 [80].

Bone resorption occurs at different rates in various body regions during space missions [79]. The bone resorption rates for the spine, neck, trochanter, and pelvis are 1.06%, 1.15%, 1.56%, and 1.35% per month, respectively, despite current countermeasures in place [81]. Conversely, bone loss is not observed at significant levels in the arm (0.04% per month) and is limited to 0.80% to 0.90% and 1.2% to 1.5% per month in the total lumbar spine and the hip, respectively [82].

Multiple studies have supported these findings, including 14% bone resorption in the proximal femur during 4 to 6 months of Mir missions [83–85]. LeBlanc et al. [81] reported that the lean arm tissue underwent no atrophy, whereas the lean leg tissue underwent atrophy at a rate of 1.00% per month. The bone recovery post-flight was reported to be slow, with approximately 2 to 3 years needed to regain pre-flight levels, and this raises serious concerns about the increased risk of osteopenia and fractures during extended space missions [28,86].

### Injuries to the musculoskeletal system

**Fig. 5.**
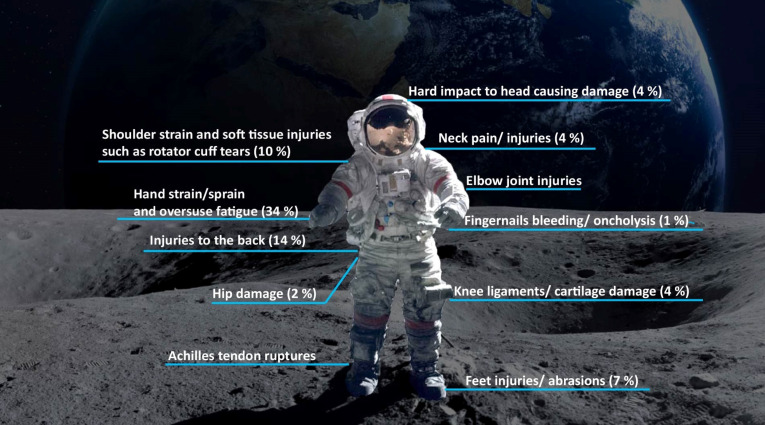
Physiological injuries and adaptations during/after spaceflights. Injury data obtained from Ref. [92]. Parts of the figure have been modified from Shutterstock.com (license: 360211214).

While the previous subsections discussed the different adaptations the human body could undergo, injuries are also a prevalent reality. Musculoskeletal injuries during space missions (Fig. 5) primarily affect the hands, back, and shoulders due to repetitive activities, locomotion, restrictive clothing, and microgravity adaptation [50,87,88]. The restrictive EVA suit, the Extravehicular Mobility Unit (EMU), is critical for spacewalks and EVA training and poses challenges to astronaut comfort and safety [87,89,90]. The EMU consists of multiple layers, starting with the liquid-cooling and ventilation garment (LCVG), which maintains thermal balance through water circulation. A rigid fiberglass component called the hard upper torso (HUT) follows this and provides structural attachment points [89].

The pressurized EMU suit hinders movement in a space vacuum, causing discomfort, fatigue, and injuries to the skin, muscles, and joints [87]. Poor EMU fit can exacerbate musculoskeletal disorders, such as microgravity-induced lower back pain [91]. With most activities relying on the upper extremities, overuse and repetitive strain injuries commonly affect the hands, shoulders, and feet [89]. Approximately 34.2% of injuries occur in the hands and 10.7% occur in the shoulders. Shoulder injuries mainly result from contact and strain at the HUT attachment points [92].

During EVA, fingertip and fingernail injuries are prevalent due to the use of pressurized gloves, substantially affecting hand strength, dexterity, and comfort [93]. Persistent exposure leads to subungual hematoma (redness), fingernail pain, and onycholysis, potentially escalating secondary infections [87]. These infections pose a notable challenge, given the decreased efficacy of medication in space and the potential for bacteria to develop resistance [87]. Other issues include fingertip abrasions, frostbite, neuropathies, dislocations, subungual hematomas, and muscle stress [87,94]. Increased moisture in the glove and reduced blood flow to the fingernail bed also contribute to these problems [87,94]. An exhaustive analysis of spacesuit glove-induced hand trauma can be found in Ref. [95].

Back injuries are the second most common injuries, after those to the hands, followed by injuries to the shoulders, feet, arms, and neck, due to crew activities and in-flight exercises [50]. The ISS has a higher incidence of back injuries than other spaceflight missions, such as Mir or the Shuttle, which is attributed to the introduction of exercise devices, including the Interim Resistive Exercise Device (iRED). While these devices aim to reduce microgravity-related physiological adaptations, they have also increased minor physical injuries, primarily strains and sprains, with contusions less common [50]. Although fractures are rare in flight, bone resorption during spaceflight increases the risk of post-flight fractures. Consequently, enhanced management protocols are needed for astronauts to exercise and prevent in-flight and post-flight injuries.

## Current Countermeasures

It is clear from the previous section that musculoskeletal problems arising from the space environment can adversely affect astronauts’ physical condition. This can also affect the success and outcomes of long-term space missions. Counteracting these effects is thus essential. Aerobic and resistive exercises have been among the earliest countermeasures proposed to mitigate the adverse effects of microgravity. These aim to increase the load on the lower extremities.

Different space agencies have been investigating additional countermeasures, ranging from specific exercise devices to artificial gravity (AG), pharmacological interventions, and nutrition. Through the Human Research Program [96], NASA has been investigating solutions that combine traditional approaches (e.g., exercise, nutrition, and pharmacological interventions) with more unconventional methods (e.g., AG, neuromuscular electrical stimulation [NMES], and vibration) [96,97].

Resistive/aerobic exercise remains the most effective countermeasure, with other methods contributing to improved effectiveness [97]. Regarding nutrition, the effects of eucaloric, hypocaloric, and hypercaloric intake, as well as protein intake management and supplementation, have been studied, along with pharmacological interventions such as bone-resorptive medications (bisphosphonates) and low-dose testosterone doping [97]. However, this review will focus primarily on physical/exercise-based countermeasures. It should be noted that while research is available on the physiological implications of different exercise countermeasures, limited information is available on the engineering specifications of the technologies discussed next. The available data are mostly from publicly disseminated information on space agency websites and as part of background sections in some literature.

### Exercise countermeasures on the ISS and other space missions

**Fig. 6.**
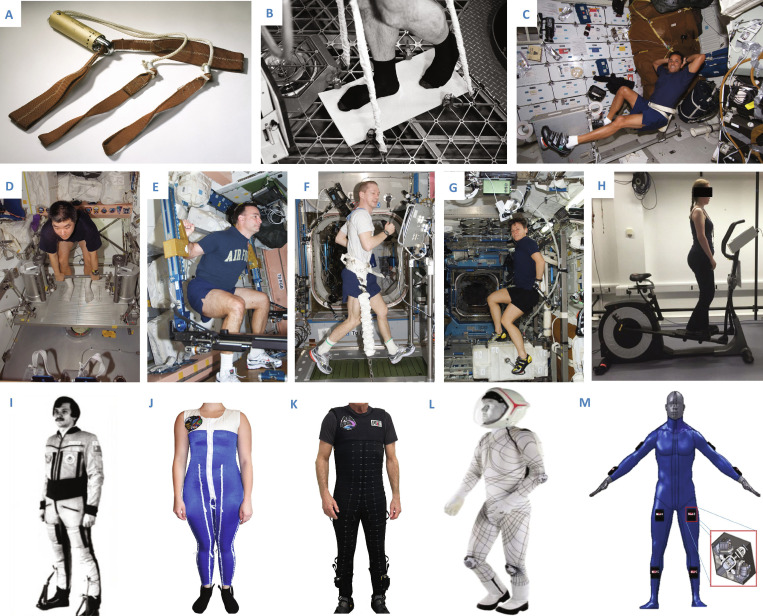
Overview and evolution of deployed and underdevelopment countermeasures. Top row: (A) An early mechanical resistance device for the apollo exerciser used on the Apollo 11 mission (photo by Eric F. Long, Smithsonian National Air and Space Museum [NASM 2009-4775] ©Smithsonian Open Access ©©Ø); (B) a Teflon-coated treadmill-like device used during Skylab 4 for aerobic exercise. Image courtesy of Skylab 4 - Treadmill-like Exercise Device - JSC by ©NASA Johnson, licensed under CC BY-NC 2.0; (C) Cycle ergometer device used onboard the Discovery space shuttle for lower-limb training. Image courtesy of ©NASA. U.S. Government work, public domain. source: Picryl. Middle row: (D) iRED device used on Expedition 16 flight. Image courtesy of ©NASA); (E) ARED device used during the space shuttle missions. Image courtesy of ©NASA; (F) COLBERT treadmill device used on Expedition 21 flight . Photo credit: NASA, image courtesy of ©ESA; (G) CEVIS device used onboard the ISS. Image courtesy of NASA, The U.S. National Archives, public domain. source: Picryl; (H) the underdevelopment FRED device. Reproduced with permission from Winnard et al. [144], ©Elsevier. Bottom row: (I) The Pingvin/Penguin (Adeli) countermeasure suit providing elastic resistive loading to mimic gravity effects. Reproduced with permission from Kozlovskaya and Grigoriev [137], ©Elsevier; (J and K) different versions of the Gravity Loading Countermeasure Skinsuit (GLCS) generating axial loading for musculoskeletal maintenance. Reproduced with permission from R. F. Bellisle and D. Newman [136]; (L) mockup of the Biosuit, a mechanical counterpressure suit designed for enhanced mobility and life support. Reprinted with permission from Porter et al. [143], ©IEEE; (M) the Variable Vector Countermeasure Suit (V2Suit) offering directional resistance for balance and neuromuscular training. Composite image using NASA public-domain imagery, with module detail image used from Ref. [145] licensed under CC-BY 4.0.

Astronauts follow a strict regimen of exercise, diet, and pharmacological supplementation to minimize bone loss and muscle atrophy. While exercise devices initially featured simple resistance bands (Fig. 6A), bungee cords, and treadmill-like devices (Fig. 6B), they have since become more sophisticated. Astronauts now spend about 2 h daily on specialized equipment such as treadmills like the Treadmill with Vibration Isolation Systems (TVIS) [98], T2, and Combined Operational Load Bearing External Resistance Treadmills (COLBERT) (Fig. 6F); stationary bikes such as the Cycle Ergometer with Vibration Isolation System (CEVIS) [99] (Fig. 6C and G); and advanced resistive exercise equipment (ARED) (Fig. 6E) [100] and iRED (Fig. 6D) to counter the effects of skeletal muscle unloading experienced in space [101]. Newer resistive and aerobic exercise devices, such as the Functional Re-adaptive Exercise Device (FRED) [102] (Fig. 6H) and SoniFRED [103], are currently under development and testing [72].

Treadmill use and higher nutrition intake in Skylab 4 reduced leg muscle atrophy [77], but bicycle ergometer and exerciser protocols did not yield remarkable benefits in preventing muscle atrophy. The iRED, which provided resistance of up to 125 kg via elastic cords, was introduced on the ISS in 2000. However, experimental results showed that it could not provide sufficient or constant resistance to prevent leg muscle atrophy or bone resorption.

Therefore, the ARED was introduced in 2009 and eventually replaced the iRED, which utilizes vacuum cylinders and flywheels, allowing astronauts to perform leg exercises, such as squats and deadlifts. The ARED substantially provided greater resistance of around 275 kg, though crew members can only exercise one degree of freedom (DOF). Smith et al. [104] reported that, upon returning to Earth, astronauts who exercised with ARED in space had a higher proportion of lean mass and lower fat mass than those performing exercise with iRED. Although ARED is more effective, it is time-consuming and has been reported to impede visual acuity.

A combination of ARED and pharmaceutical interventions (e.g., bisphosphonates) showed promise in preventing bone loss, but their long-term side effects in space are unknown [105,106]. As mentioned previously, some sprains and strains were reported during regular use of the ARED. It should also be noted that balanced mechanical systems, such as the ARED and others, unlike free weights, do not allow for the simultaneous training of stabilizer muscles. Hence, they can be less effective but also result in fewer injuries.

The FRED (Fig. 6H) has been developed to provide lumbar–pelvic reconditioning, with a focus on the lumbar multifidus (LM) and transversus abdominis (TrA) muscles, and to reduce symptoms of SABP [102]. A combined intramuscular and surface electromyography (sEMG) study by Weber et al. [107] using FRED found sustained activation of the TrA and LM muscles, endorsing its use as a countermeasure.

Despite promising results in reducing or preventing LM and TrA deconditioning, FRED has not yet been deployed on the ISS. To counter SABP, researchers have also employed virtual reality to help users achieve correct orientation and posture [108] and to provide vestibular information for restoring proprioception in microgravity.

In general, resistive exercises have been shown to help prevent bone resorption in certain areas, such as the lumbar spine and hip [83,84,109]. Rittweger et al. [109] found that resistive exercises during HDBR prevented bone resorption in the tibia but not in the radius, lumbar spine, or hip. In a study using flywheel resistive exercise during HDBR, calf muscle atrophy and bone resorption were partially reduced [109]. This resistive exercise device enabled a gravity-independent workout and was found to increase muscle volume and strength. It was further found that High-intensity interval training (HIIT), a popular protocol for alternating periods of high and low-intensity exercise, proved more effective as a countermeasure against microgravity [110]. This protocol improves neurological, cardiovascular, or musculoskeletal fitness by increasing peak muscle power, lean muscle mass, and lung capacity. The protocol has been used since the earliest space missions, but the choice of exercises, their duration, and their effectiveness are now being optimized. The intensity, frequency, type of training (aerobic or resistance), and loading are essential considerations when evaluating countermeasure technologies and protocols [111].

### Artificial gravity

AG has also been proposed as a potential solution to mitigate space-induced microgravity effects [112,113]. The Japanese Space Agency (JAXA) implemented an AG system for mice and reported some success. However, implementing a successful system for humans remains challenging due to unknown optimal parameters and limited studies supporting its efficacy [114–116]. Also, spinning an entire spacecraft is cost-ineffective and presents safety hazards, so the focus has been on short-radius centrifuge systems [112]. Creating an AG equivalent to 1G requires a substantial angular velocity (approximately 30 RPM) and could cause transitional adaptation consequences, such as vestibular disruptions [117].

### Neuromuscular/functional electrical stimulation

Functional electrical stimulation (FES) or NMES is a promising intervention that uses electrical impulses to activate muscle contractions. It provides a targeted approach to maintaining muscle mass and effectively counteracting muscle atrophy in microgravity environments. Mayr et al. [118] used an EMG-NMES system called MYOSTIM-FES to apply electrical stimulation (generating 20% of maximum voluntary force) for 6 h daily to 4 muscle groups: quadriceps, hamstrings, tibialis, and triceps surae. Although detailed results are unavailable, they found a 5% reduction in atrophy and an increase in Type 1 and Type 2 fibers. The technology was nonintrusive and user-friendly. Duvoisin et al. [119] reported improved muscle volume, mass, and CSA of twitch fibers in astronauts using the same technology. In the late 90s, NASA developed the StimMaster FES Ergometer [120] and the Percutaneous Electrical Muscle Stimulator II [121,122] used in the ISS’s human research facility.

In a recent study, FES of the triceps brachii increased muscle mass but did not significantly improve strength [123]. On-ground studies on the quadriceps group also replicated these findings [124,125]. It is hypothesized that FES/NMES may attenuate myostatin pathways that preserve mass but do not restore signaling pathways for strength [124]. FES/NMES exploration as a countermeasure is a relatively recent development, and the optimal electrical stimulation parameters remain to be investigated. FES/NMES has the advantage of providing selective muscle recovery and activating type II muscles at lower forces [126].

Recent data further support the potential of NMES as a workable defense against spaceflight-induced musculoskeletal deterioration. NMES can produce muscle contractions similar to walking-induced skeletal loading while having a significantly lower metabolic cost, as shown by Abitante et al. [127]. This implies that NMES could enhance current workout routines by providing additional mechanical stimuli throughout the day without increasing crew workload or energy consumption. These results support NMES as an operationally feasible, low-resource supplement to existing countermeasures, although further verification in microgravity conditions is needed. However, its long-term effectiveness and safety must be assessed for extended space missions. FES/NMES has been known to have limitations, such as causing rapid fatigue and difficulty in providing reliable, consistent stimulation, and has also been experienced in subjects in recent studies [126,127].

### Limitations of current exercise countermeasures

Current countermeasures are approaching functional limits, and astronauts are still returning to Earth with musculoskeletal deconditioning despite extensive exercise protocols and advanced devices on the ISS. Furthermore, excessive exercise can increase the probability of injury and generate free radicals, leading to oxidative stress. NASA and other space agencies are creating notably smaller spaceships than the ISS, making the currently bulky and extensive exercise countermeasures infeasible. For instance, the Lunar Gateway spaceship [128], developed as part of the Artemis programme, is only 12.5% the size of the ISS. Similarly, the Tiangong Space Station [129], Axiom Space Station [130], and Bigelow Aerospace B330 [131] are all future low Earth orbit (LEO) space stations that are substantially smaller than the ISS. Most space stations and spaceships have been designed for missions for 4 to 6 astronauts in a few weeks.

Secondly, while the costs of carrying loads to space have decreased since the first missions, which were over $41,000/kg during the Space Shuttle era, they are still high at over $1,700.00 per kg, even accounting for the economics of reusable rockets [132]. This would render most platforms, such as TVIS, CEVIS, ARED, and others, unsuitable for the stations. Apart from concerns about weight, bulk, and the extent of current countermeasures, there are concerns about compatible materials, electronics insulated from EMI and radiation, vibration insulation, thermal sensitivity, leveraging existing power sources (whether pneumatic or electric), and so on.

Mounting operational, structural, and logistical issues increasingly limit the effectiveness and long-term feasibility of these countermeasures on the ISS. The ISS has been the main venue for testing and applying current countermeasures created to combat musculoskeletal atrophy in microgravity [133]. However, maintaining the ISS, including these countermeasure systems and other onboard infrastructure, results in annual operational expenses of about $3 billion, with system upgrades exceeding $1 billion annually. These financial strains are further heightened by the station’s aging hardware and structural deterioration, including ongoing air leaks and microcracks in the service module of the Russian segment, which have intensified and currently present risks to both habitability and system performance.

The resulting constraints on module integrity and internal space diminish the ability to incorporate new or more advanced countermeasure technologies. Additionally, logistical challenges, especially in mass and payload capacity, further impede the delivery of innovative countermeasure equipment, shielding, or experimental apparatus. With plans already underway for ISS decommissioning around 2030, which includes an $843 million contract awarded to SpaceX for controlled orbital re-entry [133], the window for enhancing and expanding current countermeasures is closing fast. These circumstances highlight the urgency of shifting musculoskeletal countermeasure research to next-generation commercial LEO platforms, where design can more effectively meet the technical innovations and the unique spatial and operational requirements of long-duration human spaceflight. Hence, the gap between existing countermeasures and protection against musculoskeletal deconditioning requires research on novel, individual-specific countermeasures for upcoming interplanetary space missions [132].

## The Opportunities and Challenges for Wearable Technologies as Countermeasures

It is clear from the previous section that the countermeasures employed thus far are insufficient. However, a promising, relatively underexplored avenue is the development of wearable technologies specifically designed for space applications. Wearable robotics, as part of the inner or outer suit of astronauts or in the form of exosuits, is a promising alternative that can serve as a countermeasure to the musculoskeletal system’s stressors in space and mitigate their impact on astronaut physiology, overall health, and performance [134]. For example, they can act as programmable resistive exercise devices, providing continuous passive and active support to the musculoskeletal system. In addition to serving as countermeasures, these technologies can also enhance astronauts’ performance, enabling them to do tasks that are more challenging than they would typically be able to handle.

Additionally, wearable sensing can monitor astronauts’ health and their electrophysiological signals, body motion, mechanics, and interactions with their environment and suits [135]. This wealth of information can be used to warn of potential associated injuries, provide insights into astronauts’ psychosocial state and overall physical health, monitor the efficacy of countermeasures, and design better suits. Electrophysiological signals and body motion can complement each other to control wearable robotic systems.

Wearable robotics is still in its relative infancy in terrestrial applications and is not yet widely employed on Earth or in space. Aside from some limited examples in the industry, such as demonstrators or outputs of research projects, wearable robotics is not commonly used. On the other hand, wearable sensing is extensively used on Earth in its basic forms (e.g., watches, patches, jewelry, and chest bands) with limited sensing capabilities, including electrocardiogram (ECG), pulse oximetry, motion detection, sleep patterns, and breathing rate.

Advanced wearable sensing devices such as epidermal electronics, sweat analysis, and wearable microfluidics have been extensively researched for terrestrial applications. Wearability necessitates small form factors, which, in turn, require miniaturization, flexibility, and stretchability. To achieve a small form factor, highly integrated application-specific microelectronics are needed. However, such miniaturized, highly integrated, mixed-signal microelectronic components are not available as radiation-hard components. The available radiation-hard components are somewhat limited, hindering the implementation of advanced wearable devices in space. Additionally, the limited space in the shuttles and the need to use equipment or wearable suits further constrain the deployment of wearable devices in space. These technologies, holding great promise for both space and terrestrial applications as well as for human life in general, are discussed below.

### Experimental wearable technologies on space missions

Wearable systems such as the Pingvin [136] (Fig. 6I) and Gravity Loading Countermeasure Skinsuit (GLCS) (Fig. 6J and K) have been investigated to mitigate the effects of microgravity since the 1990s [137–140]. These suits, designed to emulate gravity passively via strategically positioned elastic bands or weaves, were tested on the Mir and ISS. The Pingvin suit, the predecessor to the GLCS, delivered a 0.5G load without exercise but was criticized for discomfort and inadequate thermal conductivity.

The GLCS, drawing lessons from the Pingvin suit, incorporated bidirectional elastic weaves to enable variable axial loading through tension application from the shoulders to the feet [141]. On average, the GLCS managed to impose a 0.7G load. However, it was reported to be quite restrictive and to slow locomotion, making its use on the ISS impractical. Despite these drawbacks, the GLCS demonstrated potential benefits, including improved ventilatory response, reduced perceived workload in microgravity, and decreased spinal elongation, suggesting a possible role in mitigating back pain [141,142]. Another technology being developed by the same laboratory is the Biosuit (Fig. 6L), a mechanical counterpressure suit leveraging the body’s strain fields and the concept of lines of non-extension. This design was proposed as an alternative to pressurized spacesuits and consequently reduced the discomfort and potential injuries suffered by astronauts [143].

More recent research has focused on the development of the Variable Vector Countermeasure Suit (V2Suit) (Fig. 6M) [144,145]. The V2Suit utilizes wearable modules comprising an inertial measurement unit (IMU) and a control moment gyroscope (CMG) to deliver dynamic resistance to different body segments. The IMU tracks orientation, position, and motion, while the CMG generates torque and resistance [144]. However, IMU-based orientation estimation can be unreliable in microgravity, as the lack of a 1G gravity reference causes cumulative drift and orientation errors. In response to this, a drift-resilient algorithm has been proposed [146] that maintains accurate IMU tracking without relying on gravitational or magnetic references, using local tangential and centripetal accelerations to improve robustness in 0G environments. The integration of V2Suit with GLCS and novel algorithms could enhance dynamic loading to counter muscle atrophy. However, its effectiveness depends on the algorithm’s robustness and the accuracy of the IMU’s estimations [145].

**Fig. 7.**
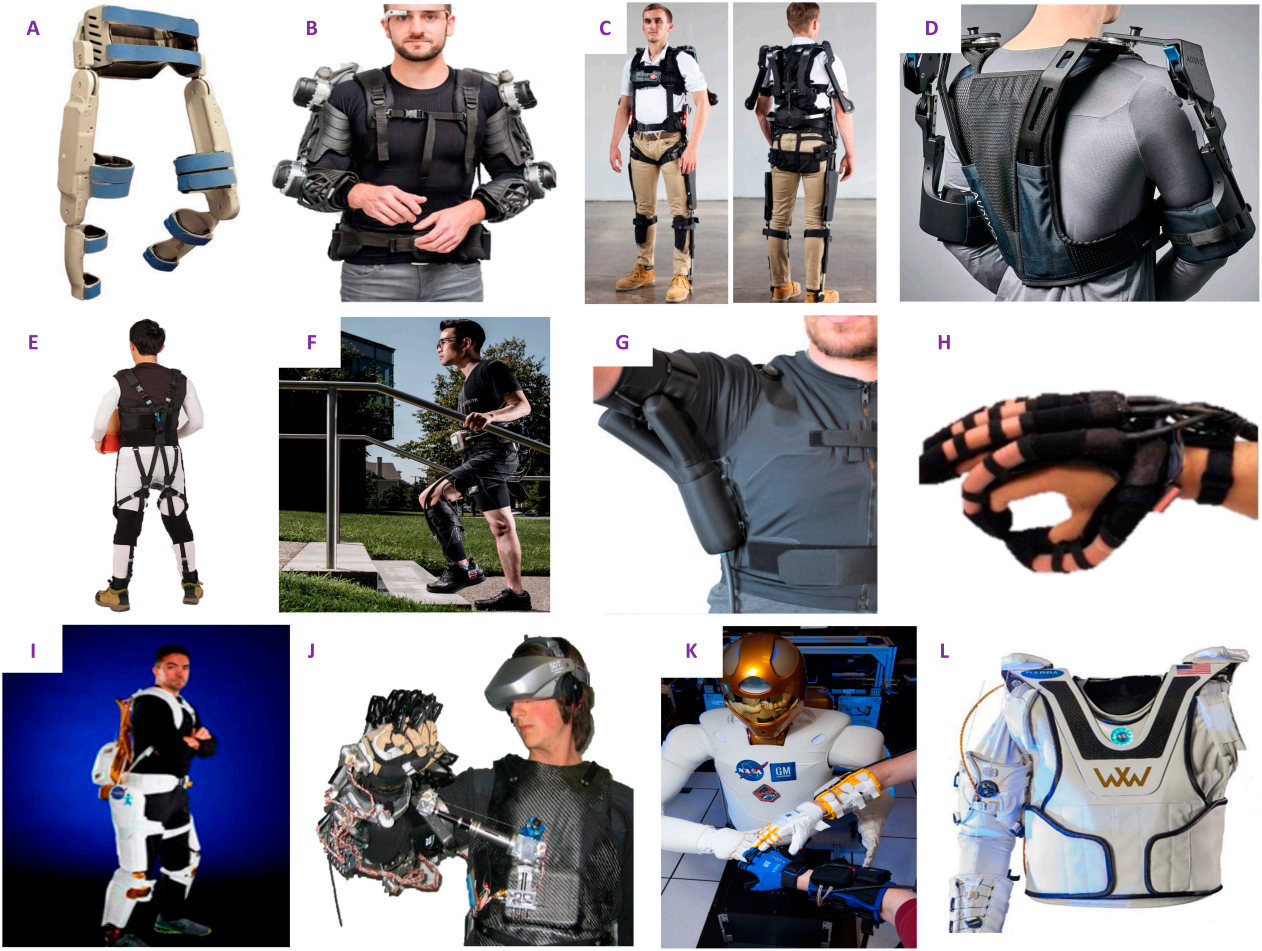
An overview of wearable robotic systems developed for terrestrial and space applications. Top row: (A) The Vanderbilt powered orthosis, now commercialized as the Indego exoskeleton, providing gait rehabilitation. Reprinted with permission from Farris et al. [189], ©IEEE; (B) the Stuttgart Exo-Jacket for upper-body support and load reduction during manual handling. Reprinted with permission from Ebrahimi et al. [158], ©IEEE; (C) the SuitX exoskeleton consisting of the shoulderX, backX, and legX components (US Bionics, now acquired by ©Ottobock) [156] providing modular industrial and medical assistance. Image courtesy of ©Ottobock/SuitX. Used with permission; ; (D) the DeltaSuit by ©Auxivo. Image courtesy of ©Auxivo. Used with permission. Middle row: (E) A body-powered variable impedance suit to reshape lifting posture. Reproduced with permission from Yun et al.[306], 6(57) 2021 ©AAAS.; (F) a tendon-driven ankle exosuit, now commercialized by ReWalk. Reproduced with permission from Malcom et al. [179], 356(6344) 2017 ©AAAS; (G) a pneumatic exosuit for restoring arm function. Reprinted with permission from O’Neill et al. [214], © 2020 IEEE; (H) a fluid-powered soft robotic glove for grasp assistance and hand rehabilitation. Reproduced with permission from Polygerinos et al. [180], ©Elsevier. Bottom row: Robots developed for space-related applications: (I) the NASA X1 exoskeleton co-developed with IHMC/NASA. Image courtesy of ©NASA [148]; (J) an exoskeleton developed at the ESA for telerobotics/telepresence applications. Image courtesy of ©ESA [149]; (K) the NASA RoboGlove co-developed with GM ©NASA for grip augmentation and hand fatigue reduction. Image courtesy of ©NASA/GM; (L) the Armstrong shoulder exosuit co-developed with Rice University for upper-limb strength. Image courtesy of ©NASA/Rice University [150].

As part of its Game Changing Development (GCD) program [147], NASA co-developed several exoskeleton/exosuit technologies for in- and post-flight exercise, rehabilitation, and assistance applications. NASA’s co-developed X1 lower-limb exoskeleton (Fig. 7I) with the Florida Institute for Human and Machine Cognition (IHMC) serves as a resistive exercise device in space and for terrestrial assistive applications [148]. The European Space Agency (ESA) and its telerobotics laboratory also developed several systems, such as the EXARM, X-ARM-II, SAM, and the ESA exoskeleton (Fig. 7J) [149]. However, these exoskeletons were developed for telepresence and haptic feedback applications rather than specifically for assistance or rehabilitation.

NASA has also embarked on developing softer systems, including the Robo-Glove (Fig. 7K) [146], a spin-off from the Robonaut 2 project, developed in collaboration with General Motors (GM), to aid in physically intensive and repetitive tasks. The Armstrong system (Fig. 7L), co-developed with Rice University, aims to enhance shoulder augmentation and rehabilitation [150]. Similarly, the Japanese and Russian space agencies have also investigated and invested in exoskeleton technologies.

These wearable systems could function as a portable gym for astronauts, providing constant muscle loading and potentially replicating gravity, thereby supplementing or replacing traditional exercise sessions. In the space-constrained ISS environment, where time and productivity are crucial, wearable devices could provide full-body monitoring, apply targeted resistance profiles for training, and serve as both assistive and resistive devices with a simple change in control strategy. As most of these systems were developed by space agencies rather than as part of academic research, detailed descriptions are not always available for a thorough review. Details of research-based and some commercial wearable robotic and sensing systems will be discussed in the next section to present their potential as alternatives to current countermeasures.

### Classification of wearable technologies by force transmission level

The potential of wearable robotics and sensing technologies to monitor musculoskeletal health and to provide an active countermeasure through dynamic muscle loading or assistance could be game-changing. In space-based applications, wearable robotic technologies could provide small forces, ranging from partial dynamic muscle loading and mimicking a sense of partial gravity to high-force active assistance during physically strenuous IVA and EVA tasks. Based on this, exoskeleton technologies can be broadly classified by the magnitude of force transmission from the robot to the wearer. The magnitude of force transmission required for the application would determine the embodiment, actuation principle, and the extent and size of the exoskeleton, as well as whether the suit can be predominantly soft/compliant, rigid, or hybrid in form factor. Low-force applications could be based on lightweight, low-form-factor soft-bodied exosuits driven by cable-based actuation, while high-force applications would use rigid frames and may even employ hydraulic or pneumatic actuation.

#### Medium-to-high force applications

Astronauts performing an EVA must wear the EMU suit, which consists of multiple layers culminating in the HUT. In the vacuum of space, this pressurized suit can severely hinder movement, requiring the astronaut to fight against both the suit and physical activity for hours, causing substantial discomfort, fatigue, and potentially skin, muscle, and joint injuries. Furthermore, EMU fit misalignment can exacerbate injuries and musculoskeletal disorders like microgravity-induced lower back pain. With most activities relying on the extremities, we observed that overuse and repetitive strain injuries commonly affect the hands, feet, and shoulders. For assisting with medium- to high-force applications, a rigid-bodied exoskeleton technology integrated into the suit is a potential solution for astronauts. In addition to assisting during IVA/EVA, these compact exoskeletons could also provide constant resistance during various activities, mimicking the effect of Earth’s gravity and/or providing constant loading, acting as a wearable gym for the astronaut.

Among exoskeleton technologies, rigid-bodied exoskeletons have been the dominant design architecture, providing a rigid frame and facilitating high force and torque transmission (Fig. 7A to D) [151–153]. These systems can be actuated actively (Fig. 7A and B) or passively (Fig. 7C and D). The same hardware platform could substantially improve performance for active systems with more sophisticated controller designs. A number of these systems have received regulatory approval and are now commercialized for various high-force/torque applications [154–156]. In terrestrial applications, lower-body systems (Fig. 7A and B) are more prevalent than upper-body systems (Fig. 7C and D), with few systems achieving complete portability [155,157,158]. The rigid-body design archetype enables high forces to be transmitted through the exoskeleton’s frame rather than through the body, as with soft exoskeletons, facilitating higher force transmission.

However, rigid-bodied exoskeletons have disadvantages, such as being heavy and extensive, requiring high-torque actuators, and large power sources [159,160]. Lightweight systems made from materials such as carbon fiber can improve wearability but may still limit the range of motion for basic ADL [161–164]. The sub-optimal mechanics of engineered rigid-bodied systems, compared to the complex biomechanics of the human body, can result in restrictive or constraining movements and be a potential source of discomfort, pain, or injury [153,165–167]. Systems designed with self-aligning [168] or self-adapting [169] mechanisms may alleviate this problem, but would come at the cost of increased size and weight [170–173]. Rigid exoskeleton design is an exercise in optimizing the size and weight of actuators, power sources, and frames, which can form a vicious cycle, and the design of heavy/bulky systems has led to the discontinuation of military projects such as HULC and XOS [151,153,174]. However, advances in power storage, materials, actuation, and sensing technologies make rigid-bodied exoskeletons increasingly realistic for real-world applications.

In space, while microgravity leads to many maladaptations in the human body, it provides an advantage for adopting rigid exoskeletons, as weight is no longer a limitation. However, the device’s bulk, size, and inertia remain the same. This allows for more sophisticated designs, powerful actuators, or heavier power sources (such as batteries or compressed fluid storage). Additionally, microgravity allows actuators to operate without having to contend with gravity, instead relying solely on inertia, friction, and other gravity-agnostic forces, thereby breaking the vicious cycle that rigid-bodied systems often suffer in terrestrial applications. This advantage in space could make rigid-bodied exoskeletons a preferred option for assisting with challenging, labor-intensive EVA activities.

#### Low-to-medium force applications

While the previous subsection discussed medium-to-high force transmission, there is also the opportunity for low-to-medium force applications. In space, this primarily pertains to technologies that can be worn for extended periods without being obtrusive or causing discomfort. The technology could act as an active wearable countermeasure, providing a sense of partial gravitational loading, and as a wearable gym, encouraging the wearer to put more effort into ADLs, helping maintain muscle tone and preventing atrophy. The GLCS and Pingvin suit aimed to achieve this passively by partially counteracting gravity’s effects. A second application of the same technology could be at-home rehabilitation post-flight.

As weightlessness is no longer advantageous in terrestrial applications, lightweight, unobtrusive, transparent, compliant, and low-profile systems could prove more beneficial. Technologies based on soft robotics principles could be ideal for this application. Wearable soft robotic systems are lighter, less restrictive (with a greater range of motion and a lower risk of injury), and more energy-efficient than their rigid counterparts [175], facilitating assistance for complex joints such as the shoulder and hip (Fig. 7E to H). Soft robotics emerged as a field as the need for enhanced physical human–robot interaction and the limitations of conventional robot design in complex environments increased [176]. Researchers drew inspiration from nature, incorporating the compliance of soft tissues [176,177] and embodied intelligence [178] into their designs.

Soft robotic principles are ideal for assisting multi-DOF joints with complex biomechanics, such as the wrist, shoulder, and hip. However, current research primarily focuses on single-DOF assistance (see Fig. 7E to H and [179–185]). Some systems demonstrate the concept of simultaneous multi-DOF aid [186–189], but only a few have demonstrated this capability [186,187]. Soft-bodied systems can have a low profile and be concealed and worn under regular clothing, thereby removing psychosocial barriers associated with advertising weakness or dependency on assistive technology. They also use less expensive, more widely available materials, thereby increasing affordability [175]. The low profile, unobtrusiveness, and improved wearability of these systems would also be advantages inside spacecraft and space stations, where real estate is at a premium. Most soft-bodied wearable robotic systems have focused on hand exoskeletons due to the low force requirements or on assisting single-DOF movements, such as the elbow. As we move up the limb, with increased mass and inertia, developing completely soft systems to achieve 100% assistance becomes more difficult, with few systems capable of multi-DOF shoulder assistance. Similarly, most lower-limb systems focus on assisting the ankle to improve propulsion, whereas hip systems are less prevalent or provide only limited assistance.

The biggest drawback of soft-bodied exoskeletons stems from their principal strength: the absence of a rigid external frame. The lack of a rigid frame results in limited force and torque transmissibility, the absence of force grounding, difficulty in achieving direct drive, and challenges in sensor and motor mounting [188].

Moreover, the limited power output from soft actuation technologies also results in lower force and torque. However, limited force generation may not be a shortcoming in space-based applications, as actuators do not have to overcome gravity to provide assistance or resistance. This could make space applications an ideal testing ground for soft-bodied systems. However, one of the biggest challenges with a low-profile, nondirectly driven system (regardless of the actuation principle) is that its low profile limits the available moment arm, consequently reducing the torque applied to the joints. While this is not necessarily a challenge for the fingers or the wrist, developing purely soft systems, such as the shoulder, becomes challenging. A potential middle ground built on hybrid systems could provide higher forces. These systems could employ rigid and soft components [190] or materials and mechanisms that stiffen and soften on demand. Another challenge in the design and implementation of soft exoskeletons is the nonlinearities arising from the compliance in soft embodiments and the influence of the user’s body on the system dynamics, which complicates controller design [191–194]. Some systems have investigated on-demand variable stiffness to overcome this shortcoming. A review of different stiffening technologies was compiled in Refs. [190,195], while a detailed review of soft robotic suits was compiled in Refs. [196,197].

#### Actuation principles for wearable technologies

Traditionally, rigid-bodied exoskeletons have used electricity or pneumatics/hydraulics as energy sources [198]. A comparison of actuator principles, including their descriptions, advantages, disadvantages, and suitability for wearable robotics in space applications, is given in Table 2. Electromagnetic actuators, such as direct current (DC) motors, are prevalent in exoskeletons due to their availability, reliability, ease of installation, operation, and control [199]. Examples of systems using motors include the Indego exoskeleton (Fig. 7A), the Stuttgart Exo-Jacket (Fig. 7B), the HAL Single Joint Elbow [154], and the Hand of Hope [200], with various transmission systems, both using direct-drive [189], and indirectly through tendons [201], linkages [200], and chain-and-sprocket [202] mechanisms. Series elastic actuators (SEAs) with sophisticated human-in-the-loop controllers are now being developed to realize individual-specific profiles that maximize assistance and aim to address challenges such as the high impedance characteristics of DC motors and other actuator-related limitations [203].

**Table 2.** Actuation principles in wearable systems

Actuation principle	Brief description	Pros	Cons	Suitability for wearable robotics in space applications
**Passive elements (springs, dampers, and gyros)** [308]	Forces provided are proportional to quantities such as displacement and velocity	Low complexity, predictable behavior, no power source needed	Lack of ability to change force profiles on demand	Passive elements are helpful for passive compliance and energy storage, beneficial in space suits for flexibility
**Motors—DC/brushless direct current (BLDC)** [309]	Electromechanical actuators for direct-drive or transmitted using cable-driven or other systems.	Reliable, controllable, compact, widely available	High intrinsic impedance (low compliance), potential safety issues during failure or spasmodic events, and higher weight compared to pneumatics	Typical for actuation in robotics, providing precise control and efficiency, but need to be lightweight and energy-efficient for wearables. It can be unsafe due to high intrinsic impedance.
**Pneumatic actuators** [222]	Use compressed air for force generation, including PAMs	Lightweight, high power-to-weight ratio	Requires air compressors/tanks, slow response time, noise, safety concerns from leaks, nonlinear characteristics, hysteresis in case of PAMs	Potential but generally unsuitable for space wearables due to reliance on compressed air, which is difficult to store and manage in space. Soft pneumatics can be considered.
**Hydraulic actuators** [222]	Fluid-driven actuators capable of providing high force	High force generation, efficient, capable of precise control	Heavy, noisy, prone to fluid leakage, complex maintenance	Generally unsuitable for space wearable robotics, as they require complex systems for fluid storage and pressure control, which are challenging in microgravity.
**Series elastic actuators (SEAs)** [310]	Motors coupled with elastic elements for compliance	High compliance, safer interaction, better shock absorption	Narrow functional bandwidth, limited use in high-speed tasks	Suitable for wearable robotics in space, as they offer compliance and adaptability, making them well-suited for delicate movements in space suits.
**Functional electrical stimulation (FES)** [311]	Elicits muscle contractions through electrical stimulation	Compact, leverages natural muscle power, and can be energy-efficient	High fatigue, discomfort, and poor controllability require sophisticated control	Potentially effective for stimulating muscle movement, making it ideal for assisting astronauts’ muscle activity in space, where muscle atrophy is a concern.
**Shape memory alloys (SMAs)** [225]	Materials that change shape upon heating or electrical activation	Compact, lightweight, simple design	Slow response time, low force generation, limited functional lifespan	Suitable for lightweight, compact actuators in space wearables, but limited by their thermal management requirements and low efficiency.
**Electroactive polymers (EAPs)** [226]	Polymers that change shape in response to electrical stimulation	Flexible, lightweight, energy-efficient, silent	Low force generation, limited durability, complex control, nonlinearities, and high voltages are required	Suitable for lightweight, flexible actuation in wearable robotics, though they may need to be optimized for space conditions like temperature and vacuum.
**Dielectric elastomers** [312]	Elastomers that deform under electrical activation	Fast response, programmable, high efficiency	Low force generation requires pre-stretching, requires high voltage, and has nonlinearities.	Suitable for soft, adaptive wearable robotics in space. They also have high energy density but require careful control.
**Twisted string actuators** [228]	Coiled nylon or other materials twisted to produce contraction	Lightweight, compact, high force-to-weight ratio	Slow response, hysteresis, complex control mechanisms	Suitable for space wearable systems, though their control systems must be precise.
**HASEL actuators** [230]	Electrohydraulic transducers with muscle-like motion	Muscle-mimetic, programmable, versatile applications	Oil retention issues, encapsulation challenges, and sensitive to temperature variations	Suitable due to their flexibility, light weight, and ability to generate high forces. They are practical for soft robotics in space.
**DLZ actuators** [227]	Dielectrophoretic actuators	High control bandwidth and power-to-weight ratio	Requires high voltage, oil retention and encapsulation, and nonlinearities	Suitable, novel, lightweight, and capable of operating under extreme conditions, they make them a potential candidate for space wearables.
**Magnetorheological/electrorheological fluids** [306,308]	Fluids whose viscosity changes under magnetic or electric fields	High adaptability, which can be used for variable stiffness	Heavy, requires active control systems, limited portability, and can only dissipate energy	Offer tunable stiffness but require an external field (magnetic or electric), which can be challenging to manage in space environments.
**Fluidic fabric muscle sheets** [232]	Flexible sheets integrated with fluid-driven actuation	Lightweight, conforms to shapes, energy-efficient	Low force generation, limited robustness	Though flexible and adaptable, it would need a reliable and compact fluid management system, which is a challenge in space applications.

Conventional pneumatic systems offer a favorable power-to-weight ratio (when pressurized containers are not included) and were developed for finger movements [204] and wrist pronation/supination [205]. These actuators have been adapted into pneumatic artificial muscles (PAMs), a different approach using pneumatics inspired by biological muscles, which have been widely used in rehabilitation and assistance applications in both rigid-bodied and soft-bodied systems [206,207]. Recently, a study demonstrated a multifunctional wearable robotic system that notably enhances user mobility and muscle activation through adaptive actuator control via PAM while maintaining minimal additional metabolic cost [206]. Building on the biomimicry approach, they have been used directly or indirectly through tendons and linkages [193,208]. These actuators have limited displacements but can exert high forces. However, like other soft actuators, they suffer from their nonlinear nature.

Hydraulics or pneumatics in space presents an additional challenge: managing large temperature variations, and hydraulic fluids can cause short circuits and other damage. Hydraulic SEAs, which combine hydraulics and electric motors, have also been used in fixed platforms, such as the NeuroEXOS system [209]. Apart from weight no longer being a limitation, pneumatic systems can be a potential actuation source, built on cutting-edge knowledge and implementations from spacecraft and space stations, and leveraging pre-existing resources, including access to compressed gases.

Soft exoskeletons [196,197] have been developed with passive (Fig. 7E) and active (Fig. 7F to H) actuation principles. While most systems were designed for terrestrial applications, they provide a comprehensive overview of the field, facilitating further research into space applications. Soft exoskeletons based on passive actuation principles can be efficient from a power/weight perspective. Systems built on top of the GLCS and other concepts leverage springs, dampers, inertia, or other novel passive elements to apply resistive forces or promote good ergonomics [141], which could be desirable for space applications. Among active actuation principles, DC/BLDC motors have been the most common method for controlling cable- or tendon-driven systems. Examples include commercial systems such as the Robotic SEM Glove [174,210] and the system by Bae et al. [179] (Fig. 7F) commercialized by ReWalk and other research-based systems [181,182,211].

Cable/tendon-driven systems with centrally located actuation packs are more common than direct-drive systems, as mounting motors on soft frames is challenging and adds weight and inertia to the limbs being assisted. Tendon-driven systems offer an easy setup, maintenance, remote actuator placement, controllability, a low profile, and higher forces compared to pneumatic and hydraulic soft actuators [181,212]. Some tendon-driven hand exoskeletons are under-actuated and use only a single actuator for multiple joints [199]. Localized forces from tendons or attachment points are a concern when using cables or tendons and require elements to improve force distribution. This challenge intensifies as force requirements increase, leading to increased cable tension, such as in systems for the shoulder or hip.

Using pneumatics and hydraulics, soft actuators ranging from single-chamber gloves [213] to more sophisticated elastomer-based actuators (with/without internal chambers and reinforcements) have been developed for achieving programmed displacements such as contraction, bending, and twisting [214–218] (Fig. 7G). Kassanos et al. [219] provided a solution to derive reinforcement configurations based on desired tip trajectories. A different design approach by Pylatiuk et al. [220,221] proposed a wearable system using an actuator inspired by spider legs. Some of these systems have been developed for at-home rehabilitation with a portable actuation pack for hydraulic fluid/air canisters and controllers [183,187] (Fig. 7H). However, compared to cable-driven systems, pneumatic and hydraulic systems suffer from limitations such as lower control bandwidth, slow response time, and restricted portability due to their tethering to air compressors or tanks (though smaller portable canisters are an option) [212,218].

Soft pneumatic actuators also face challenges, including low output forces, limited functional bandwidth in PAMs (although force output is high), noise, and safety concerns stemming from sudden pressure release due to leaks or bursts [222]. PAMs, like most other novel soft actuation methods, exhibit hysteresis and substantial nonlinear behavior, necessitating the development of appropriate control strategies, particularly to achieve accurate joint trajectory tracking. Relatively straightforward as well as more sophisticated modeling methods, including echo state networks [223], have been used to approximate the system dynamics for improved behavior prediction. Recently, hybrid models integrating pneumatic and electric actuators have been developed to combine the benefits of both—high accuracy, force capability, and back-drivability. Actuation systems must also manage extreme temperature variations, vibration isolation, radiation hardening of controller electronics, and other space-specific limitations.

Artificial actuators can be complemented by FES/NMES-induced muscle contractions to enhance control precision and reduce muscle atrophy [224]. Leveraging natural muscle power through electrical stimulation also allows for smaller, lighter systems that can be worn under clothing [126,202,207]. With ongoing miniaturization, fully wearable FES systems are now feasible. However, the use of FES/NMES in space remains limited, as the nonlinear and time-varying nature of the neuromusculoskeletal system can cause identical inputs to produce variable responses, necessitating advanced modeling and control of the human–suit system. By integrating improved artificial intelligence (AI) algorithms and predictive control, these nonlinearities can be mitigated, enabling real-time adaptation of stimulation parameters based on physiological feedback and enhancing reliability, precision, and user comfort during prolonged space missions.

Research into soft robotics continues to grow, and several soft actuation technologies for wearable robotics have been developed. Interdisciplinary research in engineering, materials, and other fields has led to novel actuation technologies such as shape memory materials [225] ionic/electronic electro-active polymers (EAPs) [226], dielectric elastomers [227], twisted nylon coil artificial muscles (twisted string actuators) [228], dielectrophoretic liquid zipping (DLZ) actuators [229], hydraulically amplified self-healing electrostatic (HASEL) actuators [230], and magnetorheological and electrorheological fluids [189,231].

The above and other concepts, such as fluidic fabric muscle sheets, liquid crystal elastomers, magnetoactive soft materials, and thermally responsive hydrogels [232], while currently at a low technology readiness level (TRL) for real-world translation in wearable robotics, could soon be incorporated into rehabilitation, assistance, and stability/support applications for both space and terrestrial applications. However, before this can be achieved, challenges and limitations need to be overcome, such as precisely modeling and controlling for the nonlinear behavior of most of these actuators, among others. Concept-specific limitations include the slow response of shape memory materials and twisted string actuators, the oil retention and encapsulation of DLZ actuators, and the low force capabilities of electroactive polymers and dielectric elastomers. The advantages and disadvantages of traditional and novel actuation mechanisms, along with their suitability for space applications, are summarized in Table 2.

### Wearable sensing technologies

Sensors that monitor the psycho-physiological state, musculoskeletal adaptations, human–spacesuit interactions, and countermeasure effectiveness are crucial to astronaut well-being and operational success while also facilitating human–robot synergy in wearable robots [2,198,233]. The strict operational demands of space agencies, such as NASA, ESA, JAXA, and ROSCOSMOS, necessitate compact, user-friendly, rugged devices with long battery life and Food and Drug Administration-approved clinical support [234].

Additionally, these sensors must withstand radiation beyond Earth’s atmosphere, requiring active electronics developed using radiation-hardening techniques and shielding, particularly within the South Atlantic Anomaly. This subsection aims to briefly discuss different sensing modalities loosely along the lines of (a) physiological effects of adaptations and injuries; and (b) biomechanical sensing and detecting user intent. This is because such sensors can be used to monitor astronaut health, assess the efficacy and impact of countermeasures, detect and predict injuries, and control wearable robotics.

#### Injury, fatigue, adaptation, and physiological monitoring

**Fig. 8.**
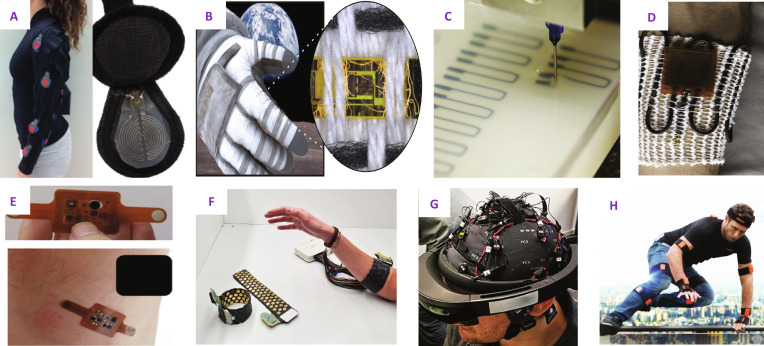
An overview of wearable sensing systems developed for terrestrial and space applications. Top row: (A) A textile-integrated, liquid metal-based resistive pressure sensor array for spacesuit dynamics. Reprinted with permission from Anderson et al. [242], ©IEEE; (B) textile-integrated InGaZnO (IGZO) thin-film transistors for space applications. Reprinted from Costa et al. [243], published under a CC-BY 4.0 license; (C) extrusion 3D printing of directly embedded resistive strain sensors. Reproduced with permission from Muth et al. [237], ©John Wiley & Sons; (D) electromagnetic resonant spiral proximity sensors for monitoring shoulder joint clearance in space suits for injury prevention. Reproduced with permission from Loftlin et al. [92], ©Elsevier. Bottom row: (E) NFC-powered flexible chest patch for fast assessment of cardiac, hemodynamic, and endocrine parameters. Reprinted with permission from Rosa et al. [247], ©IEEE; (F) a wearable high-density EMG sleeve. Reproduced with permission from Varghese et al. [307], published under a CC-BY 4.0 license; (G) subject wearing an AR headset (Microsoft HoloLens) and an EEG headset. Reproduced from Vortmann et al. [275], published under a CC-BY 4.0 license; (H) the XSens IMU Awinda suit developed by ©XSens. Image courtesy of XSens/Movella. Used with permission.

While the EMU suit and glove provide life support in outer space’s harsh environment, prolonged use often leads to fatigue and injuries, particularly to the fingertips and fingernails. The challenging environment and reduced efficacy of medications in space necessitate preventative monitoring. Sensing modalities employed for this purpose range from laser Doppler flowmetry probes to piezoresistive sensor strip arrays, humidity sensors, and thermocouples [94,235]. Additionally, multi-sensory glove-based approaches involving galvanic skin response and barometric pressure sensors have been utilized to assess skin moisture and perspiration, as well as transient pressure changes, during dynamic tasks [236]. Other approaches include the electromagnetic skin patch with a radio frequency resonant spiral proximity sensor [92], proposed for assessing the distance between the suit, the LCVG, and the skin. However, these sensors are affected by their proximity to the metal in the HUT (Fig. 8D).

Other sensing technologies for monitoring body–suit–environment interaction can measure forces ranging from tactile/haptic levels to assess discomfort and potential for injury. Composite material-based strain/pressure sensors embedded in suits are a promising sensing method, owing to their versatility and fabrication using techniques such as extrusion-based 3D printing, laser carbonization, injection molding, and/or stencil printing [219,237–241] (Fig. 8A to C). These sensors typically employ a polymer matrix and conductive filler, facilitating the sensing of suit–body–environment interactions and using the data to optimize human–suit (robot) ergonomics and controller design [233]. In Ref. [89], human–suit interaction was assessed using a pressure-sensing mat on the shoulder and custom pressure sensors along the arm.

These versatile soft sensors utilize microfluidic channels in an elastomer filled with liquid metal (gallium–indium–tin eutectic) (Fig. 7A). Radial/circumferential, shear, and normal strains could be measured, making this approach suitable for both generalized and application-specific interaction/injury monitoring and to assess body kinematics or dynamics [89,242] (Fig. 8A). Thin-film technology, based on clean room-based microfabrication techniques, has found applications in this field (Fig. 8B) due to the potential realization of high-performance, highly miniaturized, flexible, and stretchable devices, as well as the ability to co-integrate readout electronics with sensors such as strain and electrophysiological sensors [243]. Among the various technologies explored, indium–gallium–zinc oxide (IGZO)-based transistors hold great promise for the realization of flexible transistors [243]. Recently, Song et al. [244] have developed an e-skin sensing equipment that simultaneously measures temperature and pressure, enabling both injury and stress monitoring. However, it should be noted that these systems can have lower reliability or robustness and require notable improvement in TRL before they can be adopted for space applications.

In addition to being informed about the onset and extent of injuries, understanding general physical and mental well-being, as well as the impact of different maladaptations in space, is critical. Wearable multiparametric sensing systems were developed for this purpose, such as the Canadian Space Agency’s Astroskin Bio-Monitor system [245] (measuring activity level, breathing rate, blood oxygen saturation, skin temperature, ECG, and systolic BP) and a polysomnography (PSG) system for monitoring sleep quality [246] (measuring EMG, EEG, ECG, electrooculography [EOG], thoracic movements, and airflow).

Similarly, obtaining a quantitative understanding of the extent of musculoskeletal and other adaptations in near real time by measuring electrolytes, proteins, and other biomarkers, as reviewed in the previous sections, could be vital for individual-specific tuning of nutrition, supplementation, and exercise regimens. While not explicitly developed for space applications, several multiparametric sensing devices for comprehensive analysis of body fluids, such as sweat, including amperometric (monitoring metabolites like glucose), impedimetric (monitoring biomarkers such as stress hormones like cortisol), potentiometric (measuring electrolytes such as pH, calcium, etc.), and/or bioimpedance (monitoring tissue hydration, galvanic skin response, and tissue ischemia), could be further optimized and tailored for obtaining real-time feedback on musculoskeletal adaptations (Fig. 8E) [247–251]. Many of these systems are still lab-based and require real-world product development to focus more on improving robustness and reliability.

Beyond health and injury, monitoring physical and mental fatigue is crucial, particularly for mission-critical and hazardous IVA/EVA activities. Muscle fatigue, evaluated using sEMG and Mosso’s ergograph (Fig. 8F), could be particularly useful, especially during labor-intensive IVA/EVA activities [215]. Technologies integrating multiple sensing modalities, such as brain–computer interfaces (BCIs) integrated with augmented reality/virtual reality (AR/VR) (Fig. 8G), computer vision, eye-tracking, and AI, can provide not only context awareness but also real-time monitoring and adaptive instructions, drastically improving performance and quality during critical IVA/EVA tasks [252,253].

#### Sensing kinematics, dynamics, and detecting intent

Having astronauts wear wearable robotic systems solely as an exercise countermeasure will not leverage the technology’s full capabilities. If astronauts intend to use wearable robotic systems for active assistance during IVA/EVA tasks, misinterpretation/delays in understanding user intent would result in unnatural compensations, increased mistakes, frustration, and eventual disuse of the technology. Therefore, apart from the monitoring applications already discussed, sensing the body–suit–environment state, interaction, kinematics, dynamics, and intent in real time becomes vital [151,233]. The different sensing modalities, along with their advantages and disadvantages, are tabulated in Table 3.

**Table 3.** Sensing modalities in wearable systems

Sensing modality	Brief description	Pros	Cons
**Laser Doppler flowmetry probes** [94,235]	Measures blood flow velocity using laser light reflection.	Noninvasive, high sensitivity.	Requires precise positioning. Wearability, miniaturization and power consumption can be issues.
**Humidity sensors** [94,235]	Monitors skin moisture levels for perspiration assessment. Typically, they are capacitive or resistive.	Simple, effective for moisture detection.	Limited accuracy under dynamic conditions due to response-time constraints.
**Thermocouples** [94,235]	Detects temperature variations to monitor physiological responses.	High accuracy, widely used.	Slower response time in varying conditions.
**Galvanic skin response sensors** [236]	Measure skin conductance to monitor perspiration and stress levels.	Tracks psycho-physiological states.	Unreliable due to sensitivity to external environmental factors.
**Barometric pressure sensors** [236]	Detects transient pressure changes during dynamic tasks.	Compact, easy to integrate.	Limited to localized pressure sensing.
**Electromagnetic proximity sensors** [313]	Tracks skin-to-suit distance using RF resonance.	Provides spatial clearance information.	Affected by metal proximity interference.
**Thin-film IGZO transistors** [243]	Flexible, stretchable thin-film device for strain and electro-physiological sensing.	Co-integration with electronics, high miniaturization, bulk fabrication, high performance.	Limited durability under high strain, complex and expensive fabrication.
**Piezoresistive sensor strip arrays** [94,235]	Measures pressure changes by monitoring resistivity variations in strips.	Low-cost, robust, reliable.	Limited resolution for small-scale forces. Hysteresis is an issue.
**Composite strain/pressure sensors** [239–244]	Embedded in suits, measures interaction forces via polymer matrix and conductive fillers.	Versatile, customized and affordable fabrication is possible.	Complex to fabricate, hysteresis, and transiency in the sensor baseline.
**Microfluidic liquid metal sensors** [89,242]	Measures strain using liquid-metal-filled elastomer channels for normal and shear forces.	High flexibility, multi-directional.	Complex fabrication and integration into textiles.
**Textile-based strain sensors** [91]	Fabric-integrated sensors to measure posture and complex movements.	Flexible, wearable under clothing.	Limited sensitivity for fine movements.
**Tactile and resistive sensors** [241,272]	Measures interaction forces for haptic and tactile feedback in wearable robots.	Cost-effective, reliable.	Limited precision in dynamic settings.
**Capacitive force sensors** [212,219]	Measures user interaction forces by varying capacitance.	High accuracy, low hysteresis.	Sensitive to environmental changes.
**E-skin sensors** [244]	Simultaneously measures temperature and pressure for monitoring injuries and stress.	Multi-sensing capability, compact.	Requires specialized materials.
**Astroskin bio-monitor system** [245]	Monitors multiple parameters, including activity, breathing rate, blood oxygen, ECG, BP, and skin temperature.	Multi-parametric, compact system.	Radiation shielding is needed for space.
**Polysomnography (PSG) system** [246]	Monitors sleep quality by EMG, EEG, ECG, EOG, thoracic movements, and airflow.	Comprehensive physiological monitoring.	Bulky, less portable.
**Surface EMG (sEMG)** [255]	Detects muscle activation signals to measure fatigue and intent.	Noninvasive, high usability.	Motion artefacts and sensitivity to changes in surface conductivity require calibration.
**Brain–computer interfaces (BCIs)** [257,299]	Records EEG or metabolic fNIRS changes for monitoring neural intent and workload.	Enables intent detection, hands-free.	Limited real-time accuracy, noisy signals.
**Inertial measurement units (IMUs)** [300–302]	Tracks body kinematics (angle, velocity, and acceleration) in real time for motion monitoring.	Compact, widely adopted.	Signal drift over time.
**Eye-tracking sensors–AR/VR integrated** [275,276]	Tracks gaze direction for context awareness and intent detection in AR/VR uses.	Enables real-time interaction feedback.	Requires integration with headgear.

Control inputs in wearable robotics have evolved from simple analogue [195] and expiration switches [209,254] to advanced electrophysiological measurements based on EMG (Fig. 8F) and BCIs (Fig. 8G). Electrophysiological measurements can be superior, as they can even detect the onset of movement. Surface EMG is valued for its noninvasiveness, ease of use, and compatibility with wearables while offering insight into neural intent. Kuroda et al. [255] have successfully utilized myoelectrical signals generated by muscle contractions for both sensing and actuating hand control for tactile interaction. This and similar systems enable intuitive control of devices by translating natural muscle activity into mechanical motion. Advances have incorporated biomechanical and neuromusculoskeletal models for smoother control [256], high-density EMG (HD-EMG) data acquisition (Fig. 8F) [257], and novel signal-processing techniques [258–260] for intuitive control of orthotics and prosthetics. Wearable systems for monitoring muscle activation in astronauts have been developed and tested.

BCIs are gaining attention in wearable robotic control, where they record EEG or metabolic functional near-infrared spectroscopy (fNIRS) changes [261]. They have been used to control robots via motor imagery [203], visual evoked potentials [259], and P300 signals [258]; however, they have not yet demonstrated accurate real-time control. BCIs, combined with other sensing modalities like eye-tracking and computer vision, could aid in context awareness and more accurate user intent detection and cognitive workload monitoring [252] (Fig. 8G). While real-time BCI control remains a challenge, promising research efforts based on issuing higher-order commands than low-medium-level control [262], employing fast-switch-based methods [263], and predictions generated by forward models [264] are being used to inform robot movements and move closer to achieving real-time control.

In tandem with muscle and neural activity signals, various strain, force, and kinematic sensing approaches can offer vital insights into the human–robot system state, interactions, and user intent, thereby regulating controller input [184,201,264–266]. Joint angle, velocity, and acceleration [218,267] measurements are frequently employed, occasionally in conjunction with joint torque metrics [268,269]. The widespread use of IMUs (Fig. 8H) in various real-world systems, including space-based wearables, underscores their effectiveness [144,145,187,270]. Textile-based wearable sensors provide an innovative alternative for monitoring kinematics and physiology. Combined with statistical models, neural networks, or other AI-based algorithms, they enable the accurate prediction of complex body movements, such as torso, lumbar shape, and posture [91], as well as multi-DOF movements [271].

For intuitive human–robot interaction, force-sensing technologies are essential for understanding dynamics and the interactions among humans, robots, and their environment. They provide valuable metrics to controllers, utilizing tactile [196,209], capacitive [212,219], and resistive sensors [241,272] to track user intent and generate appropriate movements and forces. Inductive [273] and deformation-based sensors, based on load cells, monitor interaction forces, providing a key input to the wearable robot’s closed-loop controller [274]. Conductive composites, liquid metals, and other strain/pressure sensing modalities [219,237–242] (Fig. 8A and C). As discussed in the previous subsection, this approach could also be used for sensing interaction and obtaining tactile or haptic feedback. Context awareness, utilizing data from cameras and eye trackers, which can be potentially integrated within AR/VR headsets (Fig. 8G), when combined with neural and muscle activity signals, could help identify user intent more accurately [275,276].

### Challenges for wearable technologies as countermeasures for space missions

Human spaceflight involves the interplay among physiological, environmental, and external stressors, which impact astronaut health and operational performance. We identified microgravity, radiation, and psychosocial stressors [4] as the most prominent connected challenges facing astronauts in spaceflight. These challenges will likely degrade the body’s capacity to maintain functional strength and coordination and also limit cognitive resilience and emotional stability. We argued that in response to such challenges, wearable robotic countermeasures may emerge as potential solutions to aid human physical rehabilitation and enhance task performance during long-duration spaceflight. However, there are challenges or bottlenecks to consider when utilizing these technologies in tandem with the existing complexity of human spaceflight. A thorough understanding of the interaction between spaceflight stressors and technological constraints (Table 4) is essential for developing effective, context-appropriate countermeasures.

**Table 4.** Stressors, bottlenecks, challenges, and solutions

Stressor	Bottleneck	Challenge	Gap in research	Research solutions
**Microgravity** **Precision actuation in variable gravity**	Actuators designed for 1G gravity may not function optimally in microgravity.	Creating actuation algorithms that adapt torque and stiffness profiles according to the environment.	Insufficient actuators that can adjust to different gravitational conditions.	Actuators featuring flexible control strategies that adjust performance according to different gravities.
**Real-time, adaptive control systems**	Dynamic and unpredictable external conditions in microgravity.	Introducing controllers capable of handling nonlinearities, hysteresis, and delays independently.	Insufficient models for control systems operating in microgravity.	AI-based predictive controllers and hybrid impedance-admittance controllers dynamically adjust assistance or resistance based on user intent and task needs.
**Radiation** **Radiation-hardened embedded systems and electronics**	Traditional electronics are vulnerable to ionizing radiation, which may cause failures.	Creating resilient control and signal processing hardware with fault-tolerant systems that reduce size and power consumption.	Need for lightweight, radiation-resistant electronics created for wearable systems.	Investigate advanced materials and radiation-hardened electronics to ensure reliability in high-radiation environments.
**Precision-driven, quick-response sensor integration and accuracy**	Radiation exposure, temperature fluctuations, and vibrations can impair the accuracy, performance and sensor timings.	Achieving real-time integration of sensors with processors built to withstand radiation and improve reliability.	Limited knowledge of sensor effectiveness in microgravity and radiation conditions.	Develop radiation-tolerant sensors and algorithms that compensate for errors and sensor drift by applying kinematic constraints and sensor fusion to ensure information integrity.
**Psychosocial stressors user comfort and compliance**	Wearables can lead to discomfort, stress, or decreased compliance in astronauts.	Creating devices that ensure comfort, simplicity of use, and psychosocial acceptance during missions.	Insufficient research on long-term comfort and psychological effects.	User-centered design, conduct astronaut feedback missions, and incorporate adaptive training to address psychosocial responses.
**Social isolation and confinement**	Effects of device usage on mental health.	Tackling the mental impacts of isolation on device acceptance and adherence.	There is a need for interventions to mitigate the effects of isolation on astronauts.	Wearable systems for social interaction and mental stimulation and provide psychosocial support for missions.

#### Microgravity-dependent challenges

Microgravity fundamentally disrupts human biomechanics, driving rapid physiological deconditioning. The absence of gravitational loading leads to muscle atrophy, particularly in the antigravity muscles of the lower limbs and trunk, bone demineralization, impaired proprioception, and altered sensorimotor control. These physiological changes justify the development of wearable robotic countermeasures capable of generating controlled, artificial loading during movement or exercise. However, this absence of gravity complicates the design, control, and user perception of these devices. Traditional resistive mechanisms, reliant on fixed reference points (such as ground contact), do not function as expected in microgravity. Therefore, exoskeletons must generate internally consistent force loops, through harnesses, counteracting actuators, or structural constraints, to simulate meaningful resistance. These design adaptations increase mechanical complexity and demand sophisticated actuators, sensors and control algorithms that remain stable under variable force dynamics and user input.

As human exploration extends beyond LEO, wearable systems must function in diverse gravitational environments. The moon has about one-sixth of Earth’s gravity, while Mars has roughly one-third. Each environment introduces unique biomechanical, locomotion and control dynamics (see the “Human locomotion and movement” section), necessitating real-time adaptability in assistive or resistive force profiles. Verdel et al. [277] found that humans can rapidly adjust their motor behavior; the human motor system does not merely counteract gravity but instead leverages it to reduce muscular effort while moving. However, minimizing effort would increase muscle atrophy. Therefore, predictive and optimal controllers are necessary to optimize under different gravity conditions. Otherwise, those controllers might overreact on Mars, while a controller designed for Martian movement could perform poorly in lunar conditions or in orbit. These gravity-dependent behaviors complicate traditional control paradigms, highlighting the need for adaptive control systems that can learn and adjust to the user’s motion patterns and environmental feedback. Additionally, adapting to different gravity conditions, such as transitioning from a spacecraft to a planet’s surface, requires improved sensor durability and a more adaptable controller.

In terrestrial applications, technological advancements have remarkably improved exoskeleton control systems by incorporating AI. Previously, traditional controllers required manual adjustments to accommodate different tasks and environments, which created challenges in dynamic scenarios such as space exploration [278]. The rise of AI-driven, task-agnostic controllers has shifted this paradigm by enabling real-time adaptation across various activities without requiring task-specific programming beforehand. For example, Molinaro et al. [279] demonstrated a deep neural network-based controller that rapidly estimates lower-limb joint moments, enabling exoskeletons better to assist users in a wide range of movements. Such AI methods can predict joint motions and torques across various gravitational environments and transitions, allowing them to adapt and generate compensatory forces to counteract the effects.

In summary, gravitational variability is a dynamic variable that must be explicitly integrated into control logic, sensing strategies, and actuator frameworks. Systems that fail to adapt appropriately may reduce astronaut mobility, increase the risk of injury, or compromise the effectiveness of in-flight countermeasures. As such, developing gravity-responsive robotic wearables represents a foundational challenge for planetary mission readiness.

#### Radiation

Chronic exposure to space radiation, including GCRs and solar particle events, can cause single-event effects and presents a critical, though not immediately visible, threat to astronaut health and the integrity of wearable robotic systems. The potential for radiation to damage semiconductor components, degrade sensor accuracy, and induce actuator drifts and errors in digital control systems is a concern that cannot be overlooked. Wearable robotics must be engineered utilizing radiation-hardened materials and fault-tolerant architectures, particularly those dependent on high-performance processors, micro-electro-mechanical systems sensors, and wireless communication protocols. There has been the use of radiation-hardened microprocessors for space equipment, such as BAE Systems RAD750 [280] or by Honeybee Robotics [281]. However, it is not yet modified for use in wearable robotics in space. This necessity imposes constraints on component selection, escalates power and thermal management requirements, and complicates efforts to miniaturize the system.

Additionally, the challenge of shielding wearable devices is exacerbated by the requirement that protective layers must be both lightweight and practical, necessitating that any increase in mass be warranted within the broader context of system design. In contrast to fixed spacecraft infrastructure, wearable systems maintain continuous contact with the human body, thereby requiring considerations for thermal regulation, biocompatibility, and radiation resistance. Such compounded requirements increasingly burden engineering efforts, particularly on long-duration missions beyond LEO, where radiation exposure risk is higher.

#### Psychosocial dependent challenges

Wearable technologies designed for space missions face substantial longevity issues. These setbacks include the physical heft, the challenges of donning and doffing, and the cognitive load on the user during operation. Astronauts will not adopt every biomechanical system if it negatively affects their daily lives or changes their perception of comfort. This effectively hinders the use of wearable sensorimotor or musculoskeletal health countermeasures in space. An example is the GLCS, which, while it offers advantages, was scrapped on the ISS due to discomfort, restricted range of motion, and donning challenges. These IBs affect the compliance of the astronaut population and demonstrate that comfort, usability, and user experience must be considered alongside the physiological benefits.

Conversely, soft exosuits, as potential countermeasures, offer clear benefits for user experience. These suits may enhance mobility and reduce mechanical intrusiveness, thereby decreasing the physical and psychological burdens associated with extended wear and potentially increasing compliance during long missions. However, soft exosuits are still in their infancy, with limitations in actuation precision, force output, and long-term material durability. This highlights the need for a carefully considered design strategy that balances engineering performance with behavioral and psychosocial viability. Achieving this balance requires a multidisciplinary, user-centered approach, informed by empirical insights from on-the-ground environments, such as parabolic flight experiments. These ground studies offer valuable insights into how human factors interact with wearable systems in conditions that simulate the sensory deprivation, social isolation, and variable workload characteristics of space travel.

The advancement of wearable robotics for space missions will likely depend on incorporating adaptive, intelligent technologies that can respond to the user’s physiological and psychological conditions. Wearables equipped with biofeedback sensors to monitor heart rate variability are being used on the ISS [282]. These can be tailored to measure muscle and cognitive fatigue, thereby adjusting resistance levels or training methods. This customized approach can enhance therapeutic outcomes and accommodate fluctuations in stress and motivation.

Furthermore, responsiveness becomes even more critical due to the increasing autonomy of crews and the limited support from ground control during extended deep-space missions. The successful integration of wearable robotics into astronauts’ daily routines will be essential for their effective performance. When these technologies are viewed as supportive extensions of the body rather than just tools, they are more likely to be accepted as integral parts of astronauts’ lives in space, which, to date, can be a challenge. Thus, future designs should incorporate insights from neuroergonomics, behavioral psychology, and user-centered design aesthetics. This integration will ensure that wearable technology promotes both musculoskeletal health and psychological resilience and also meets the daily needs of crew members, thereby mitigating psychosocial stressors.

Wearable devices face numerous challenges in their practical application in space. Continuous data collection can lead to information overload for astronauts and their ground control, diverting attention from detecting critical health indicators. Privacy concerns may influence comfort and, ultimately, acceptance of ground control monitoring astronauts’ health data. The technical challenges of these devices may be raised by microgravity and high radiation levels. Bulky or uncomfortable devices can interfere with an astronaut’s routine, hindering their practical use and limiting their psychosocial health applications. Addressing these pressing challenges will require a multidisciplinary approach, beginning with surveying human and ethical factors and engineering advances. User-centric design and the successful integration of wearables into astronauts’ daily lives will ultimately enhance the acceptability and uptake of monitoring devices in space exploration.

#### Other challenges

The previously discussed stress factors exacerbate the technical challenges in creating wearable robotic systems. Optimizing weight, size, and power supply remains a challenge. Traditional high-torque exoskeletons are bulky and require a relatively large amount of energy. However, this creates a dilemma: increased actuator strength requires a larger power supply, adding more weight to the system. While microgravity alleviates concerns about weight, factors such as launch mass, device inertia, cost, and energy consumption still impose limitations. Consequently, energy efficiency becomes increasingly critical, prompting research into regenerative actuators, advanced battery technologies, and innovative energy-harvesting techniques. Additionally, control algorithms must be capable of adjusting to changes in movement dynamics and decreased proprioceptive feedback, necessitating robust sensor fusion from drift-sensitive IMUs and varied EMG signals. Closed-loop control systems that respond to biomechanical data and the user’s intentions are also essential for exoskeletons during dynamic exercise.

When factoring in human elements, wearable devices for stressful situations must ensure comfort, adaptability, ease of use, and support for additional mission tasks. Rigid exoskeletons can cause discomfort due to pressure points or joint misalignments; this discomfort may arise from individual anatomical differences or prolonged wear. While soft exosuits address these issues, they can hinder force transmission and precise motion control. Reliability and safety are critical in space missions, as failure of an actuator or control system poses large risks, especially if medical staff cannot promptly fix the devices. This situation demands redundancy, passive safety measures, and fail-safes within the control systems. Beyond safety and reliability, the design of wearable technologies must ensure they do not disrupt the spacecraft’s cabin layout, emergency protocols, or life-support systems. Moreover, the device should work with over-diagnostic tools, be remotely monitored when inactive, and withstand the mechanical and thermal extremes of space travel.

## Discussion and Perspectives for Future Research and Development

As humanity begins a new era in space exploration, pursuing a sustainable human presence on the Moon and preparing for a future Mars mission, it becomes increasingly evident that existing physiological countermeasures will not suffice. Multiple threats associated with both microgravity and additional exposure to potentially harmful stressors (e.g., radiation or psychosocial stressors) present a multifaceted threat to astronaut health and performance. While decades of research have generated valuable knowledge and countermeasures, the next generation of “countermeasures” must become embedded, intelligent, and adaptive, not bulky and resource-intensive, to enable effective engagement. Wearable robot systems are a notable technology most closely aligned with these characteristics. This review is intended for researchers and designers aiming to develop the next generation of wearable countermeasures to mitigate the harsh stressors of outer space.

Current research in space physiology, particularly in musculoskeletal adaptations, provides valuable insights into bone density loss, muscle atrophy, and systemic physiological changes resulting from prolonged exposure to microgravity. Studies conducted pre- and post-flight, especially those leveraging sophisticated imaging modalities and biochemical assays on the ISS, have laid a strong foundation for understanding these complexities. While countermeasures such as resistive exercise, nutritional strategies, and pharmacological interventions have shown promise in mitigating these adaptations, they remain only partially effective. They are often bulky, time-intensive, and resource-demanding. These limitations pose substantial challenges for future missions aboard compact spacecraft. Exercise is the cornerstone of current countermeasures, with systems such as CEVIS, TVIS, iRED, and ARED installed on the ISS and in earlier ships, including Mir and Skylab. Novel solutions, including AG, are also under evaluation. However, weight, size, and payload capacity constraints demand sophisticated, lightweight, modular, and compact alternatives.

Wearable systems represent a natural evolution and opportunity in countermeasure design. By incorporating actuation, sensing, control, computation, and material innovation, they offer a path to continuous, personalized support without an operational burden. Wearable systems can function as smart “second skins”, monitoring physiological markers, providing mechanical loading, assisting locomotion, and adapting in real time to the astronaut, notably reducing the adverse effects of space stressors. While systems such as the Pingvin suit, the GLCS, and NASA’s soft exosuit prototypes provide some proof of principle for certain aspects of our vision, the development of operationalized, high-TRL, space-rated systems is in its infancy [283]. Yet, trends in advanced materials science, advanced quantum computing, soft robotics, and AI indicate that we are on the verge of a leap in capability, provided that research efforts remain focused and interdisciplinary.

Wearable countermeasures must be lightweight, thermally stable, and radiation- and mechanical-fatigue-resistant. A critical enabler for these systems is advanced materials science. Innovations in materials are crucial for advancements in battery technology, space suit design, sensors, actuators, and electronics. Therefore, multifunctional materials, such as hydrogenated polymers for radiation shielding, boron nitride nanotubes, graphene composites, and wide-bandgap semiconductors like silicon carbide, offer solutions that combine mechanical strength with electronic and radiation-resistant properties [13,284,285]. Notably, these materials must also support integration with flexible electronics, sensors, and actuators, and provide a platform for building human–machine interfaces, controls, or AI without compromising comfort or mobility. Research into soft, self-healing materials and conductive textiles may further enable distributed sensing and actuation across garments or suits [286,287]. Spacesuit embodiments must also address thermoregulation, pressure management (mechanical counterpressure versus pressurized suits), comfort, dexterity, and ergonomics. In particular, smart materials, such as SMA and EAP, with closed-loop control strategies combined with AI, can provide adjustable stiffness, as studied in soft wearables, to leverage their self-healing properties and adapt to different G environments [288].

Additionally, research into ultrathin soft radiative-cooling interfaces (USRIs) combines high solar reflectance with nearly perfect mid-infrared emissivity. This setup achieves over 56 °C of passive cooling [289], representing a remarkable advance in thermal management and long-term wearability. By potentially integrating USRI layers with radiation-hardened composites and microfluidic loops, we can create lightweight, self-optimizing exosuits that resist radiation. These suits maintain comfort and performance during lengthy space missions, marking an important step toward fully adaptive robotic enhancement for space exploration. Building on these developments, intelligent soft-robotic systems now include low-power neuromorphic circuits. These circuits may adapt to metabolic load, thermal stress, and radiation damage.

Specific sensing and actuation requirements must be prioritized to successfully implement wearable technology in space missions. The sensor-actuator loop functions as the nervous system of any wearable robotic platform, particularly in space applications where human–robot integration must be both seamless and adaptive. A coordinated array of biomechanical, electrophysiological, and biochemical sensors can provide real-time, context-aware data on movement, exertion, fatigue, hydration, musculoskeletal deconditioning, and early detection of injury risk—information critical for decision-making, responsive actuation, and mission success. Emerging sensing modalities offer promising opportunities to address these limitations. Electrochemical, myoelectric, kinematic, and environmental sensors are increasingly being explored for their ability to deliver rich, multimodal data streams [290,291]. These sensors enable advanced physiological monitoring, enhance human–robot interaction, and control adaptability. Access to high-fidelity data from these sources is crucial for making dynamic decisions, reconfiguring systems, and providing user-centered feedback, especially under the stresses of spaceflight.

Additionally, soft robotics and soft sensing technologies offer exciting opportunities to enhance wearability, flexibility, and human compatibility [292]. These systems are especially appealing for their ability to conform to the body, respond to natural movement patterns, and reduce mechanical impedance. However, several barriers still hinder their deployment in high-TRL applications [292,293]. Key issues include the nonlinear behavior of soft materials, precise control and dynamic modeling, high-voltage requirements for actuation, durability limitations, and slow response times [293]. Addressing these challenges is vital for transitioning from prototypes to practical systems.

Parallel to these sensing challenges, actuation technologies also require substantial refinement. Current approaches, including dielectric elastomers, soft pneumatics, electrohydrodynamic systems, and tendon-driven soft robotic actuators [294], offer various benefits but often fall short regarding reliability, scalability, and energy efficiency. Many existing actuators are either too complex mechanically or underpowered to be integrated into compact, wearable forms suitable for space. Designing actuators that can operate precisely and reliably in the vacuum of space, under radiation stressors, and with minimal power requirements remains an urgent area of research [295].

Neuromusculoskeletal modeling and adaptive control algorithms will be equally vital, enabling systems to interpret sensor data contextually, anticipate user intent, and coordinate actuation with natural movement across variable gravity conditions [294,295]. A multidisciplinary approach incorporating advancements in sensor technology, actuation techniques, materials science, and control engineering is essential to successfully integrating wearable robotics with the human body in space.

In support of design development, AI offers opportunities to simulate materials, mechanisms, and biological systems, enabling faster verification and validation to achieve the high TRL anticipated. Astronauts’ extensive pre-, during, and post-flight testing has generated a wealth of data that could be used to create highly accurate digital twins, both neuromusculoskeletal and other simulation systems that model the outer-space stressors [291,296]. These twins could represent various physiological systems and adapt in real time using data from onboard sensors. AI-powered systems could analyze these data and serve as decision-support systems for predictive and reactive medical/diagnostic interventions, enabling personalized exercise regimens, adaptive controllers for robotic systems, and astronaut training and education. As a result, this may improve human–machine interaction, help finely tune nutrition and pharmacological protocols, and lead to better cognitive and physical health. Moreover, when deployed on ultra-low-power neuromorphic processors [297], these AI systems can operate autonomously onboard spacecraft, supporting astronauts even in communication-limited environments such as Martian missions. This makes this research area vital for developing and testing new systems and their robust deployment on space missions.

Beyond assistance or therapy, modeling the interactions between humans, robotic suits, and the environment can pave the way for providing haptic, tactile, and other forms of biofeedback for psychosocial well-being, regular maintenance, and IVA/EVA tasks. These advancements could substantially reduce physical strain, mitigate cognitive overload, and enhance task performance. This is particularly important for astronauts, as the loss of haptic feedback and dexterity, especially when wearing pressurized gloves during EVA [298], is well-documented. Research into mechatronic systems capable of delivering such feedback can create a more natural user experience. From the cognitive perspective, employing multiple sensing modalities, such as BCI, can provide a highly accurate assessment of mental acuity, fatigue, and cognitive load, helping to prevent accidents [257,299].

Hence, human–robot interaction, both physical and cognitive, remains a foundational challenge. For wearable systems to be adopted and relied upon, they must feel intuitive and minimally intrusive. Advances in shared control paradigms, in which robotic systems modulate their behavior based on physiological signals such as HD-EMG, ultrasound, IMUs, EEG, fNIRS, and other sensing modalities, could facilitate intuitive, natural control [300–302]. Coupling these signals with AI-based intent recognition can enable seamless co-adaptation between the astronaut and the system. At the same time, haptic and tactile feedback mechanisms can restore lost sensory perception and prevent spatial disorientation, particularly during EVA activities that rely on gloved interfaces with limited dexterity and feedback [303]. Realizing a wearable robotic system that integrates all these advancements could lead to a future where astronauts achieve superior physical and cognitive performance while wearing minimalistic wearable robotic systems that feel as natural as clothing. Such systems would not only optimize their physical health and mental well-being during missions but also provide lasting benefits after mission completion.

Despite the promise of wearable systems as advanced solutions for musculoskeletal issues, the shift from ground-based prototypes to operational flight systems has been limited. This is primarily due to the unique challenges encountered during initial launches and the stresses experienced in space. Key obstacles include exposure to high-energy radiation, changes in gravity, and the psychological complexities of isolated and confined missions. Each of these factors introduces additional technical, physiological, and human-factor challenges that must be addressed to secure the safety, usability, and effectiveness of wearable countermeasures for future orbital and interplanetary missions. The identified stressors are somewhat interconnected constraints that outline the limitations of current Earth-based wearable robotics intended for musculoskeletal countermeasures during space exploration.

Another critical area needing attention is the optimization of exercise protocols for wearable robots utilized by astronauts. What is the most effective way for them to use these devices? Should they wear them continuously with light resistance throughout the day, known as the “continuous countermeasure” approach, participate in short bursts of high-intensity workouts, or use a combination of both methods? Some studies suggest that incorporating continuous low-level resistance into daily activities may preserve muscle conditioning as effectively as rigorous gym workouts [304]. Hence, if additional research supports this idea, it could usher in a profound transition from traditional, scheduled exercise to a more integrated approach to fitness in daily life, which wearable technologies can facilitate. Nevertheless, extensive research is crucial to confirm the long-term effects on bone health and cardiovascular fitness associated with these approaches.

Furthermore, most space physiology research has been conducted in LEO, limiting the applicability of learnings to extended missions or deep-space environments. Therefore, a key question is how accurately we can model and validate the wealth of knowledge and information from LEO to the deep mission. Additionally, limited sample sizes, disparate methodologies, gender imbalances, and an overall lack of generalizability among findings from different space agencies need careful consideration. Establishing standardized research frameworks and addressing these biases will be essential for advancing countermeasure development. Unlike academic research, space research is not widely disseminated, making it challenging for private companies and academic researchers to access the latest technologies and contribute more effectively. It should be a policy focus area.

To optimize the efficacy of wearable robotic systems for spaceflight applications, it is imperative to prioritize an integrated, adaptable, and human-centered design approach. Future research must examine environmental, physiological, and psychosocial robustness rather than isolating components. Subsequently, these factors must be considered at the system level for accurate modeling, such as digital twins or AR/VR. Furthermore, the system’s radiation resilience must be assessed comprehensively, considering not only individual components but also the entire system—first, the spacecraft, and second, the wearable systems—particularly when EVA is required. For prospective exosuits, it is essential to incorporate fail-operational modes within distributed control systems and garment-integrative radiation shielding, and implement error-correcting codes within all embedded processors. Additionally, advancements in materials science are crucial for developing radiation-tolerant elastomers and composites for soft actuators and structural frames.

Space R&D has historically driven innovations with far-reaching terrestrial applications, ranging from medical devices, early computers, and instrumentation to navigation and telecommunications, as well as everyday technologies. Wearable countermeasures developed for astronauts could translate into systems for at-home rehabilitation, personal fitness, assistance for the elderly, and the sustenance of life in extreme environments. By addressing the challenges astronauts face, researchers can also develop solutions that enhance physical and cognitive abilities, benefiting diverse populations on Earth. In space and during missions, wearable systems could monitor astronauts’ health, movements, and the mechanical forces they exert on their environment or on their suits. Wearable sensors could predict, prevent, or detect early injuries during critical missions. Wearable robots can assist astronauts in completing challenging tasks or counteracting the effects of the space environment and weightlessness. Sensors and robotics can be combined to improve robot control, machine learning, sensor fusion, and other techniques.

Integrating multiple disciplines presents ongoing challenges. One review notes that exoskeletons function best within a “synchronized multidisciplinary effort” [283], essential for successful designs that merge human biomechanics, robotics, and ergonomics. In the context of space, this means that engineers, exercise physiologists, orthopedic specialists, and astronauts themselves will collaboratively design suitable solutions. A user-centered approach that involves extensive astronaut feedback and prototypes that accommodate various body types will be vital to addressing astronaut requirements. Closing the cultural gap between robotics engineers and life scientists will arise from a common goal of ensuring astronaut health without excessive strain. Lastly, wearable robots complement other countermeasures, including but not limited to nutritional supplements, pharmacological agents such as bisphosphonates, promising approaches to prevent bone loss in space, and a possible AG protocol. The challenge remains in articulating how an exoskeleton regimen can enhance these methods. For instance, if an astronaut uses medication for bone loss, can the robotic suit adjust the load accordingly?

In conclusion, wearable robotic countermeasures blend space medicine and robotics in a promising way. Although there are notable challenges—such as the weight of the hardware, power supply, human-centered design, and safety—there are also lessons from Earth-based exoskeletons that can create valuable innovation opportunities, drawing on their successes and failures. Transforming an idea into a “flight-ready astro-exosuit” will necessitate closing research gaps through thorough testing explicitly focused on astronaut-centric, user-driven design. However, the potential benefits are immense; if each astronaut’s movement can double as exercise, we can keep them in a stronger and healthier state during long missions that may last decades between planets. The developments in astronaut missions to achieve this goal will also enhance wearable robots for rehabilitation and industrial use. With continued collaboration across our diverse disciplines, we might witness the first astronauts engaging in Human Lifecycle Health research, equipped with a groundbreaking supportive exoskeleton, within the next 10 years—an evolved version of a spacesuit designed not only for human survival but also to optimize musculoskeletal health throughout interplanetary voyages.
